# Late first trimester circulating microparticle proteins predict the risk of preeclampsia < 35 weeks and suggest phenotypic differences among affected cases

**DOI:** 10.1038/s41598-020-74078-w

**Published:** 2020-10-21

**Authors:** Thomas F. McElrath, David E. Cantonwine, Kathryn J. Gray, Hooman Mirzakhani, Robert C. Doss, Najmuddin Khaja, Malik Khalid, Gail Page, Brian Brohman, Zhen Zhang, David Sarracino, Kevin P. Rosenblatt

**Affiliations:** 1 Division of Maternal-Fetal Medicine, Department of Obstetrics and Gynecology, Brigham and Women’s Hospital, 75 Francis Street, Boston, MA 02115 USA; 2 Channing Division of Network Medicine, Department of Medicine, Brigham and Women’s Hospital, Boston, MA USA; 3 NX Prenatal Inc., Louisville, KY USA; 4 Department of Pathology, Center for Biomarker Discovery and Translation, Johns Hopkins University School of Medicine, Baltimore, MD USA; 5 Division of Oncology, Department of Internal Medicine, University of Texas Health Science Center at Houston, McGovern Medical School, Houston, TX USA; 6 Thermo Fisher Scientific, Cambridge, MA USA

**Keywords:** Biomarkers, Diseases, Molecular medicine, Pathogenesis

## Abstract

We hypothesize that first trimester circulating micro particle (CMP) proteins will define preeclampsia risk while identifying clusters of disease subtypes among cases. We performed a nested case–control analysis among women with and without preeclampsia. Cases diagnosed < 34 weeks’ gestation were matched to controls. Plasma CMPs were isolated via size exclusion chromatography and analyzed using global proteome profiling based on HRAM mass spectrometry. Logistic models then determined feature selection with best performing models determined by cross-validation. K-means clustering examined cases for phenotypic subtypes and biological pathway enrichment was examined. Our results indicated that the proteins distinguishing cases from controls were enriched in biological pathways involved in blood coagulation, hemostasis and tissue repair. A panel consisting of C1RL, GP1BA, VTNC, and ZA2G demonstrated the best distinguishing performance (AUC of 0.79). Among the cases of preeclampsia, two phenotypic sub clusters distinguished cases; one enriched for platelet degranulation and blood coagulation pathways and the other for complement and immune response-associated pathways (corrected *p* < 0.001). Significantly, the second of the two clusters demonstrated lower gestational age at delivery (*p* = 0.049), increased protein excretion (*p* = 0.01), more extreme laboratory derangement (*p* < 0.0001) and marginally increased diastolic pressure (*p* = 0.09). We conclude that CMP-associated proteins at 12 weeks’ gestation predict the overall risk of developing early preeclampsia and indicate distinct subtypes of pathophysiology and clinical morbidity.

## Background

Preeclampsia is exclusively a human affliction with no naturally occurring mammalian models^1^. This observation suggests that the origins of the condition lie in the unique aspects of human placentation and gestational adaptation. Rather than a single disease entity, it is more likely to be a syndrome with a core set of common features with multiple associated subtypes^2,3^. Recent work has begun to identify patterns to suggest subphenotypes of preeclampsia beyond the conventional early versus late-onset distinction^4–6^.

The syndromic nature of preeclampsia may be the reason why effective prophylaxis and therapy have been elusive^2^. Except for aspirin, many trials of physiologically plausible agents have frustratingly failed to prevent preeclampsia^7–10^. This dilemma is understandable given that it will be difficult to find a single prophylactic agent for a syndrome with potentially diverse pathologic underpinnings. Studies would be biased toward null findings just as would any attempt to find one treatment to prevent all forms of cancer. We, therefore, seek to both identify women at risk of preeclampsia as well as to characterize the sub-type of preeclampsia for which the patient is at risk.

Circulating microparticles (CMPs) are nano- to micron-sized lipid bilayer extracellular vesicles that, while only recently discovered, have been conserved by evolution and are present in all eukaryotes^11–13^. Within the human body, CMPs are released by a diverse variety of cell types including platelets, endothelial cells, erythrocytes, trophoblasts, lymphocytes and a variety of malignant cell types. CMPs have been isolated from an even wider variety of fluids including saliva, plasma, amniotic fluid, ascites, semen and cerebrospinal fluid^13–15^. Based on their size, CMPs can be divided into three categories; exosomes (50–150 nm), microvesicles (100 nm–1 µm) and apoptotic bodies (200 nm–5 µm)^16^ and contain a variety of molecular cargo that act as mediators of cellular function, including mRNA, microRNA, DNA fragments, as well as cytosolic, integral membrane, and membrane-associated proteins^15,17,18^. CMPs offer a minimally-invasive means to “biopsy” tissues at the time of secretion^19–22^.

In a murine model, CMP-mediated effects have been implicated in the modulation of maternal physiology during pregnancy^23,24^. In human gestation, the concentration of CMP increases more than two-fold during pregnancy^14,25^. Work from our lab suggests that variation in CMP-associated proteomic profiles in the late first trimester is associated with an increased risk of spontaneous delivery prior to 35 weeks gestation^26–28^. Placental CMP characteristics are already known to be altered in pregnancies complicated by preeclampsia^29–34^. Analysis of CMP composition may, therefore, offer insight into the later risk of preeclampsia^34,35^, and given the latent quality of preeclampsia physiology, this may be possible at an early stage of gestation. Such an analysis early in pregnancy also has the potential to separate preeclampsia phenotypes among those at risk of the disease.

The present study has two parts. In the first part, we hypothesize that CMP-associated proteins sampled from maternal plasma at the end of the first trimester would differ in pregnancies destined to be complicated by preeclampsia when compared to those that remain normotensive. In the second part, we hypothesize that, among cases of preeclampsia, circulating CMP proteins can be classified into different groups that correlate with maternal clinical characteristics. For both parts of this analysis, we sampled CMPs from the maternal circulation at a median of 12 weeks’ gestation. As such, the origins of these particles is a combination of maternal and placental. We seek not to distinguish the origin of these particles but to demonstrate the use of CMP associated proteins as markers of PE risk and classifiers of PE subtype.

## Results

### Sample composition and characteristics

We initially selected and processed 25 cases and 50 controls for analysis. However, after the initial sample processing had been completed, access to detailed patient medical records identified one case with HIV that had been incorrectly classified as HIV negative; in an additional case, a patient was found to be on an immune-modifying medication for a chronic autoimmune condition. These two cases were excluded in an a priori fashion from the sample classification analysis. We thus analyzed 23 cases and 50 controls.

The characteristics of the sample set are presented in Table 1. Median maternal age, pre-pregnancy BMI, and race of the cases and controls did not differ significantly (*p* < 0.05). Similarly, the percent nullipara, married, and tobacco use during pregnancy were also statistically similar in both groups. Consistent with the known risk factors for preeclampsia, the incidence of chronic hypertension and preeclampsia in a prior pregnancy was higher among the cases^36^. Neither case or control group had prepregnancy histories of renal disease or chronic proteinuria. The median gestational age at plasma sampling was similar but, consistent with the study design, the cases were delivered at a significantly earlier gestational age. Predictably, the birth weight was greater for the controls but the birthweight Z-score for gestational age was similar in both groups. The ratio of male-to-female infants was higher, but not significant, among the preeclampsia cases. Median maximum systolic and diastolic blood pressures were both significantly greater among the cases than controls. The cases had a median of 581 g per 24-h protein collection. Comparable information was not available for the control group as testing was not clinically indicated. Among the cases, the median gestational age of diagnosis was < 34 weeks (Table 1).

**Table 1:** Population characteristics.

Characteristic	Preeclampsia (N = 23)	Controls (N = 50)	*p* value^a^
	Median (IQR) or N (%)	Median (IQR) or N (%)	
Maternal age	32.3 (28.1–36.6)	31.3(26.2–36.9)	0.63
Maternal race			0.74
Black	6 (26.0%)	14 (28.0%)	
Hispanic	2 (8.7%)	10 (20.0%)	
White, non-Hispanic	15 (65.2%)	26 (52.0%)	
Nullipara	5 (22.0%)	14 (28.0%)	0.42
Smoking	4 (17.4%)	10 (20.0%)	0.79
Married	16 (70.0%)	31 (62.0%)	0.89
Chronic hypertension	3 (13.0%)	0 (0.0%)	0.009
Chronic renal disease/proteinuria	0 (0.0%)	0 (0.0%)	NA
Pre pregnancy BMI	24.6 (21.1–29.1)	25.5 (22.5–30.0)	0.74
PE in prior pregnancy	6 (33.3%)	3 (8.3%)	0.02
Prior spontaneous PTB	12 (66.7%)	8 (22.2%)	0.001
**Gestational age (weeks) at**			
Sampling	11.3 (8.7–13.4)	11.1 (9.6–13.7)	0.97
Delivery	34.4 (32.0–35.3)	39.4 (38.9–40.4)	< 0.001
**Birth metrics (g)**			
Birth weight	2215 (1400–2811)	3353 (3140–3725)	< 0.001
Z-score	− 0.24 (− 1.1–0.6)	− 0.21 (− 0.7–0.8)	0.45
**Infant gender**			
Female	15 (65.2%)	26 (52.0%)	0.29
Male	8 (34.8%)	24 (48.0%)	
**Clinical characteristics**			
Median maximum systolic (mmHg)	168 (147–180)	112 (103–120)	< 0.001
Median maximum diastolic (mmHg)	96 (88–99)	68 (61–73)	< 0.001
Median 24 h protein (gram)	581 (343–921)	NA	NA
Week of PE diagnosis	32.4 (29.7–33.0)	NA	NA

^a^*p* values calculated with Wilcoxon Rank Sum test, ANOVA, Chi Square test, or Fisher Exact test where appropriate.

*NA* not applicable/available.

**Figure 1:**
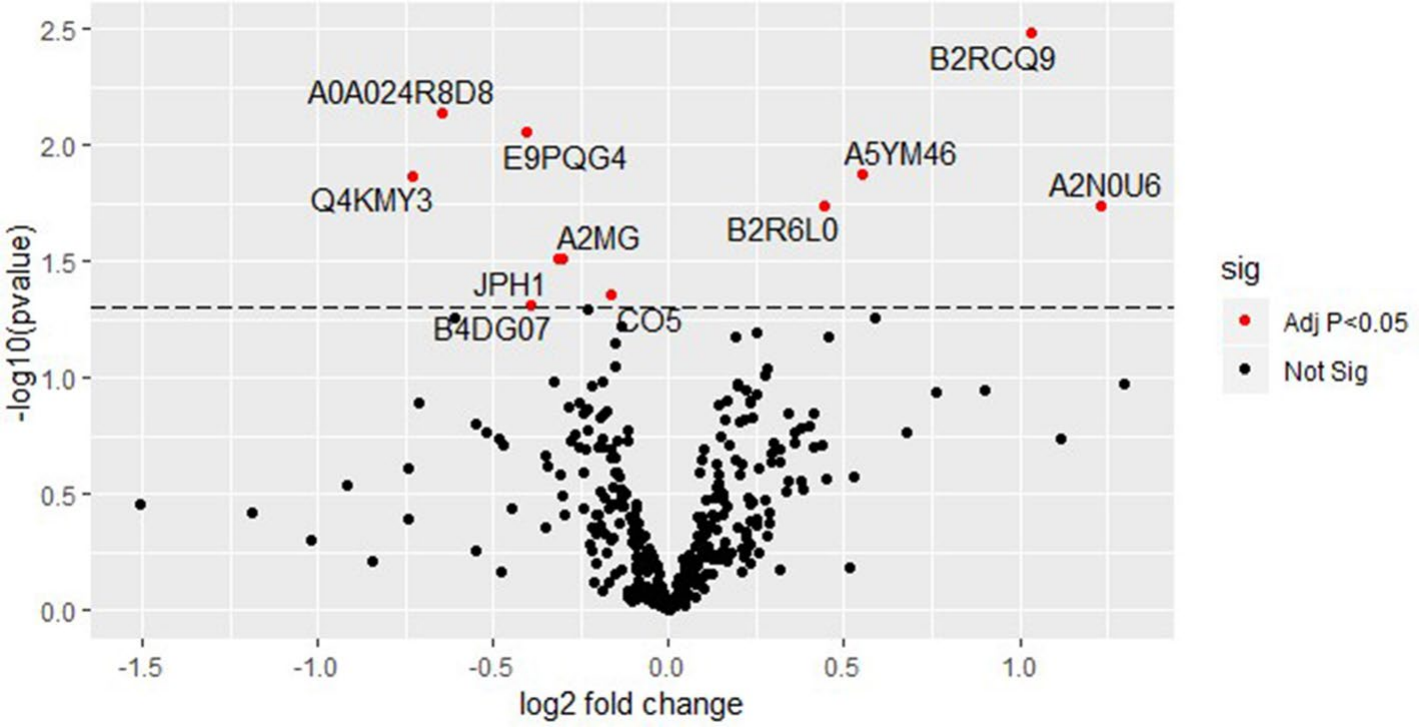
Circulating microparticle proteins associated with preeclampsia at median 12 weeks gestation. Red dots represent CMPs that were significantly associated with preeclampsia at an adjusted *p* value < 0.05. Black dots represent CMPs that were not significantly associated with preeclampsia at an adjusted *p* value < 0.05.

Signal processing using the Pinnacle Software version 1.0.92.0 (Optys Tech Corporation, Philadelphia, PA; see methods section) identified 226 proteins (Supplement Table S1). Using a bivariate comparison for association with preeclampsia (cases) in the full dataset, the proteins were ranked by *p* value adjusted for multiple comparisons (Fig. 1). Eleven proteins with an adjusted *p* < 0.05 for comparison in case versus control with their associated biologic functions and gene names as documented in the UniProt database (www.uniprot.org, accessed 9/16/2019) are listed in Table 2^37^.

Table 2 suggests, given the unreviewed annotation and unclear function of several proteins, that there is a risk of persistent false discovery. Therefore, as described, we proceeded with the restricted dataset that included proteins with ≥ 4 documented functional associations (edges) as identified in the STRING database. This restricted the initial set of candidate proteins to those with prior annotation and known biologic interactions. Using the workflow described above, we sought panels of markers associated with preeclampsia rather than a single analyte.

**Table 2:** Bivariate comparison of differentially expressed proteins by preeclampsia status.

Abbreviation	Name	Biologic function	Gene	Adjusted *p* value^a^
B2RCQ9^b^	Highly similar to Homo sapiens heat shock 70 kDa protein 1	Unknown	N/A	0.0033
A0A024R8D8^b^	Progestogen-associated endometrial protein	Small molecule binding	PAEP	0.0073
E9PQG4^b^	Myomegalin	Unknown	PDE4DIP	0.0089
A5YM46	ERN2 protein	Protein kinase activity	REN2	0.013
Q4KMY3^b^	C10orf28 protein	Unknown	C10orf28	0.014
B2R6L0	Tubulin beta chain	Cytoskeleton structure	N/A	0.018
A2N0U6^b^	VH6DJ protein	Immunoglobulin heavy chain variable region	VH6DJ	0.018
A2MG	Alpha-2-macroglobulin	Protease inhibition	A2M	0.031
JPH1	Junctophilin-1	Forms junctional membrane complexes	JPH1	0.031
CO5	Complement C5	Complement activation	C5	0.044
B4DG07	Highly similar to RAB6-interacting protein 2	Unknown	N/A	0.048

^a^*p* values calculated with bivariate logistic regression. Bonferroni correction for multiple testing.

^b^Annotation unreviewed—protein information inferred.

**Figure 2:**
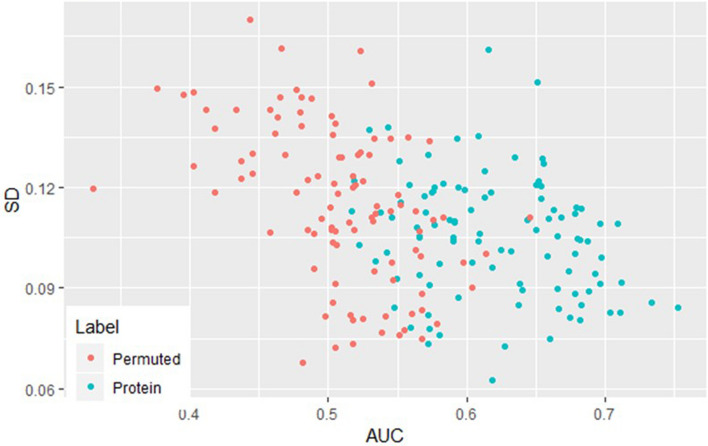
AUC versus standard deviation for protein versus permuted. Blue dots represent actual protein AUC and SD. The red dots represent AUC and SD from randomly permuted the sample labels (preeclampsia versus control).

**Figure 3:**
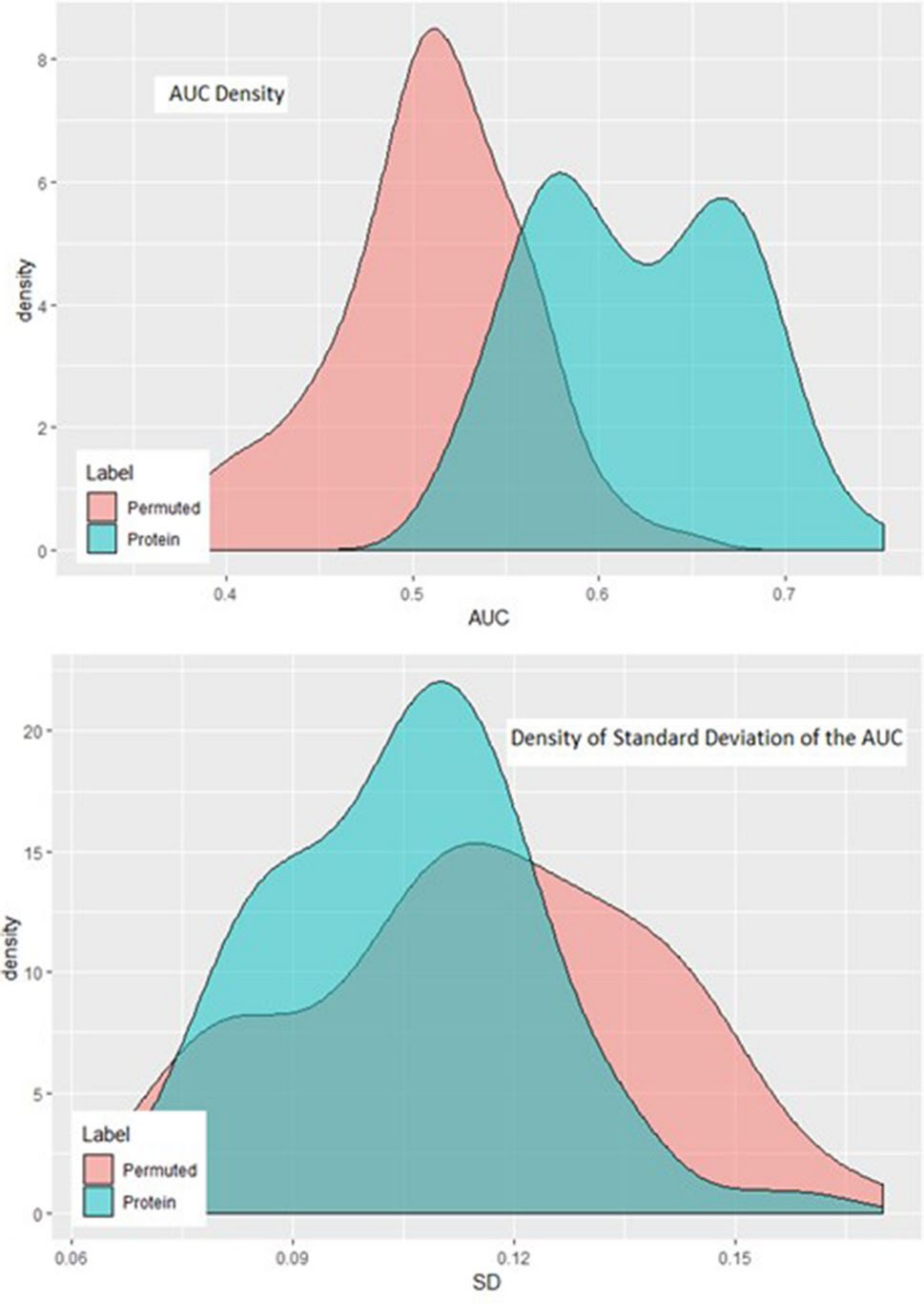
Density plots of protein versus permuted. Blue shaded area represent actual protein AUC and SD. The red shaded area represent AUC and SD from randomly permuted the sample labels (preeclampsia vs. control).

To further characterize the validity of the marker panel, we randomly permuted the sample labels (preeclampsia vs. control) and re-applied the statistical workflow. The median and standard deviation of the outer AUC for the observed and permuted panels are compared in Fig. 2. The individual observed markers have a clear tendency for higher mean AUC and a lower standard deviation of the AUC (right lower quadrant) when compared to the permuted samples. The median AUC for all the observed proteins in the panels was significantly greater than those in the permuted samples (0.62 vs. 0.51, *p* < 0.0001). Consistent with its random origins, both the permuted AUC and the standard deviation of the permuted AUC have more central and normal-appearing distributions, whereas the identified proteins have asymmetric density plots (Fig. 3). The higher median AUC and the asymmetry in the density plots suggest that these markers contain predictive information.

Table 3 lists the 17 individual proteins that reoccurred as a member of the multiple panels in at least five or more iterations. These proteins are ranked by the frequency with which they were members of the highest-scoring panel. These proteins were enriched in 26 biological processes including blood coagulation and hemostasis (N = 5), restoration of injured tissue (N = 6), and complement activation (N = 4; all corrected *p* < 0.05). Thirteen out of 17 proteins (76.5%) were enriched in the cellular compartments of extracellular exosomes and micro-vesicles (corrected *p* < 0.001). Of these, 4 proteins (4/13 = 30.77%) demonstrated enrichment in placenta (corrected *p* = 0.027; Supplemental Table S2).

**Table 3:** Most frequently recurrent individual proteins distinguishing preeclampsia from controls in 100 multiplexed panels.

Protein	Protein names	Biological function	Gene name	Frequency (%)
GP1BA^a^	Platelet glycoprotein Ib alpha chain	Blood coagulation	GP1BA	79
VTNC^a^	Vitronectin	Cell adhesion/migration	VTN	57
C1RL^a^	Complement C1r	Complement activation	C1RL	49
ZA2G^a^	Zinc-alpha-2-glycoprotein	Cell adhesion	AZGP1	46
APOC2	Apolipoprotein C-II	Cholesterol homeostasis	APOC2	37
APOH	Beta-2-glycoprotein 1	Blood coagulation	APOH	30
JPH1	Junctophilin-1	Calcium ion transport	JPH1	28
CO5	Complement C5	Complement activation	C5	16
HEP2	Heparin cofactor 2	Blood coagulation	SERPIND1	16
TPC11	Trafficking protein complex subunit 11	Golgi organization	TRAPPC11	14
MBL2	Mannose-binding protein C	Complement activation	MBL2	11
AACT	Alpha-1-antichymotrypsin	Acute-phase response	SERPINA3	8
DYH3	Dynein heavy chain 3, axonemal	Cilium-dependent cell motility	DNAH3	7
TSP1	Thrombospondin-1	Cell adhesion	THBS1	7
CAPS1	Calcium-dependent secretion activator 1	Exocytosis	CADPS	6
APOD	Apolipoprotein D	Angiogenesis	APOD	5
LCAT	Phosphatidylcholine-sterol acyltransferase	Lipoproteins metabolism	LCAT	5

^a^Constituents of highest scoring panel.

Among all combinations, the panel of markers with the highest mean AUC (0.79) and lowest mean standard deviation of the AUC (0.12) contained the following 4 markers: complement C1r subcomponent protein (C1RL), platelet glycoprotein 1b alpha chain (GP1BA), vitronectin (VTNC), and zinc-alpha-2-glycoprotein (ZA2G) (Table 3). This combination of CMP-associated proteins occurred in 11% of the iterations. The next most frequent combination included a similar constituent set of analytes, but with beta-2-glycoprotein 1 (APOH) replacing ZA2G. This second combination occurred in 3% of the iterations.

### Clustering within the preeclampsia cases

**Figure 4:**
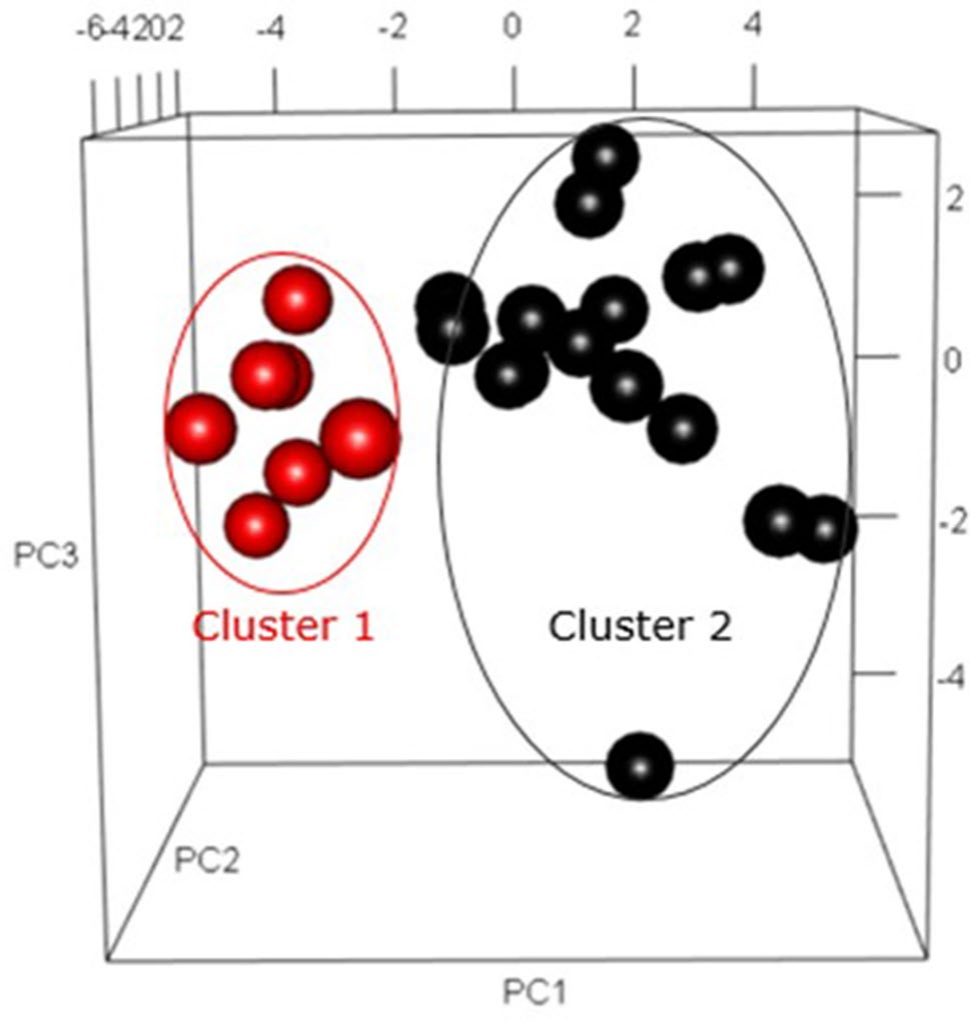
K-Means clustering of cases of preeclampsia delivering ≤ 35 weeks gestation. Red dots represent observed cluster 1 of cases, while the black dots represent cluster 2.

To examine the potential for CMP-associated proteins to identify possible subgroups of disease among the subjects with preeclampsia, we performed unsupervised clustering using the K-means algorithm that was restricted to the cases in the prior analysis. Given the limited sample size, the candidate proteins for this clustering exercise were restricted to the 31 analytes that occurred in one or more of the iterated panels in the prior analysis. Preliminary silhouette analysis indicated that the optimal number of clusters for the restricted CMP protein data was two. The K-means procedure then identified a smaller cluster of 7 (Cluster 1) and a larger cluster of 16 (Cluster 2) among the subjects with preeclampsia. Figure 4 presents the two clusters projected on the first three principal components.

Functional enrichment analysis using the g:Profiler package^37^ suggested that the proteins associated with the cases in Cluster 1 displayed biological process enrichment in pathways related to platelet degranulation and intrinsic pathway of blood coagulation (N = 3 and 2, *p* = 0.02 and *p* = 0.03, respectively; Supplemental Table S3). The molecular function was enriched for protease binding and extracellular matrix structural constituents with multiple cellular components enriching for extracellular matrix and secretory granule function (all corrected *p* < 0.05; Supplemental Table S3). The proteins associated with the cases in Cluster 2 were enriched in biological pathways for immune system responses, inflammatory and complement pathways (N = 13, N = 11, and N = 10, respectively, all corrected *p* < 0.05). Associated molecular functions tended to be centered on enzymatic activity. The cellular components associated with proteins in Cluster 2 related to blood microparticles, circulating microparticles and extracellular vesicles (N = 11, 18 and 18, respectively, all corrected *p* < 0.05). Proteins characteristic of cases in Cluster 2 also demonstrated tissue enrichment in placenta in the analysis of tissue specificity using g:Profiler (N = 8, *p* = 0.049; Supplemental Table S4).

The 22 CMP-associated proteins with an unadjusted *p* value < 0.05 for differential expression in one of the two clusters are presented in Table 4 with their functional associations as annotated in the UniProt database^38^. Five of the proteins had higher expression in Cluster 1, whereas 17 had higher expression in Cluster 2. After correction for multiple testing, vitronectin, pigment epithelium-derived factor, complement C4-A, and prothrombin retained significantly different expression between the two clusters.

**Table 4:** CMP-associated proteins with differential expression between the clusters with protein functions.

Abbreviation	Name	Function	Gene	Associated cluster	*p* value	Bonferroni correction
IGJ	Immunoglobulin J chain	IgA/IgM binding	JCHAIN	1	0.012	0.372
ZN251	Zinc finger protein 251	RNA transcription	ZNF251	1	0.022	0.697
ECM1	Extracellular matrix protein 1	Angiogenesis	ECM1	1	0.027	0.845
CD5L	CD5 antigen-like	Lipid synthesis regulator	CD5L	1	0.047	1.453
A2MG	Alpha-2-macroglobulin	Coagulation	A2M	1	0.055	2.022
VTNC	Vitronectin	Cell adhesion/migration	VTN	2	8.16E−06	0.0003
PEDF	Pigment epithelium-derived factor	Angiogenesis inhibitor	SERPINF1	2	9.79E−05	0.003
CO4A	Complement C4-A	Complement activation	C4A	2	0.0005	0.017
THRB	Prothrombin	Blood coagulation	F2	2	0.001	0.033
B4E1Z4	Highly similar to Complement factor B	Highly similar to Complement factor B	n/a	2	0.004	0.109
CO3	Complement C3	Complement activation	C3	2	0.004	0.109
ANGT	Angiotensinogen	Angiotensin-activated signaling pathway	AGT	2	0.004	0.109
CO2	Complement C2	Complement activation	C2	2	0.005	0.142
PHLD	Phosphatidylinositol-glycan-specific phospholipase D	C-terminal protein lipidation	GPLD1	2	0.005	0.143
FA12	Coagulation factor XII	Coagulation	F12	2	0.015	0.462
CFAH	Complement factor H	Complement modulation	CFH	2	0.018	0.569
CAPS1	Calcium-dependent secretion activator 1	Vesicle exocytosis	CADPS	2	0.025	0.777
CRP	C-reactive protein	Acute-phase response	CRP	2	0.027	0.845
KNG1	Kininogen-1	Coagulation	KNG1	2	0.027	0.845
HEP2	Heparin cofactor 2	Coagulation	SERPIND1	2	0.033	1.020
HEMO	Hemopexin	Heme metabolic	HPX	2	0.039	1.221
PRG2	Bone marrow proteoglycan	Immune response	PRG2	2	0.047	1.453

The population and medical history characteristics of the two clusters did not differ significantly (Supplemental Table S5). Tables 5 and 6 present the clinical characteristics of Clusters 1 and 2. The unadjusted median week of delivery was lower whereas the median maximum systolic and diastolic pressures, as well as the median 24-h protein collection at diagnosis, are all significantly higher for Cluster 2 (Table 5). After correction for multiple testing, the week of delivery and urine protein remained significant. Table 6 presents other clinically relevant parameters in the setting of preeclampsia. Cluster 2 had significantly higher proportions of subjects with extreme analyte values for creatinine, fibrinogen, and plasma sodium levels in the unadjusted testing. These values do not remain significant after adjusting for multiple testing. However, the more severe analyte values in Table 6 are consistently associated with Cluster 2. Using a permutation algorithm, we can reject the null hypothesis that the observed is a purely random association with a single cluster (*p* < 0.003).

**Table 5:** Differential pregnancy characteristics between clusters 1 and 2.

Cluster (N)	Median week at delivery	Median maximum systolic (mmHg)	Median maximum diastolic (mmHg)	Median 24 h protein (g)
1 (N = 7)	35.2	158	80	310
2 (N = 16)	33.6	170	98	726
*p* value	0.013	0.045	0.025	0.0026
Bonferroni correction	0.049	0.18	0.099	0.010

**Table 6:** Hematologic differences between clusters 1 and 2.

Cluster (N)	Creatinine > 1.0 mg/dL N (%)^a^	Fibrinogen > 450 mg/dL N (%)^b^	Sodium < 132 Mmol/L N (%)	Uric acid > 5.7 mg/dL N (%)	ALT > 50 U/L N (%)	AST > 50 U/L N (%)	Platelets < 100,000 N (%)
1 (N = 7)	0 (0.0%)	0 (0.0%)	0 (0.0%)	3 (43.0%)	1 (14.0%)	1 (014.0%)	1 (14.0%)
2 (N = 16)	8 (53.0%)	6 (66.0%)	8 (75.0%)	8 (50.0%)	2 (13.0%)	2 (19.0%)	4 (25.0%)
*p* value	0.02	0.02	0.02	0.08	0.91	0.80	0.57
Bonferroni correction	0.11	0.11	0.17	0.53	> 1.0	> 1.0	> 1.0

^a^Creatinine values not available on 1 subject in cluster 2.

^b^Fibrinogen values not available on 6 subjects in cluster 2.

## Discussion

We have defined a set of CMP-associated proteins from 10 to 12 weeks gestation that are able to stratify women at risk for preeclampsia < 35 weeks. We further identified two clinically-relevant clusters with differences in CMP-associated protein expression among the preeclampsia cases. Specifically, with regard to the former, we identified a panel of four CMP-associated proteins (C1RL, GP1BA, VTNC, ZA2G) that return potentially clinically useful information for the prediction of preeclampsia diagnosed at < 34 weeks. In the second portion of this analysis, we use CMP proteins in unsupervised clustering analysis of preeclampsia cases to expose potentially diagnostically useful sub-structure among these cases of early-onset preeclampsia. The two clusters differ in their median gestational age at delivery, parameters of morbidity and overall clinical severity, while demonstrating differences in median expression levels of multiple CMP-associated proteins.

### Characteristics of CMP-associated proteins as markers of preeclampsia at a median of 12 weeks

The proteins that distinguished preeclampsia cases delivering < 34 weeks gestation, a gestational age range considered to represent, “early” preeclampsia^39–41^, from healthy pregnancies were enriched in the placenta and annotated to functional pathways including hemostasis, blood coagulation and restoration of injured tissue. C1RL is believed to be expressed in the placenta in addition to the maternal liver and kidney^42^. These organs are commonly involved in preeclampsia pathology. Functionally, C1RL is a serine protease active in the endoplasmic reticulum, where it is involved in the proteolytic cleavage of haptoglobin^43^. It inhibits complement-mediated cytotoxicity^42^. GP1BA is one subunit of a glycoprotein heterodimer found on the surface of platelets. This protein has not been reported to be associated with adverse pregnancy outcomes. Vitronectin (VTNC) is an abundant glycoprotein in human serum and the interstitial matrix. It plays a role in both cell adhesion and migration. VTNC is involved in the binding of complement, heparin, thrombin-antithrombin III complexes, as well as the regulation of the plasminogen activation system^43–46^. Alterations in plasma VTNC concentrations have been associated with preeclampsia^47,48^. Decreased plasma levels of zinc-alpha2-Glycoprotein (ZA2G) in the first trimester have been observed in pregnancies complicated by early-onset preeclampsia^49^. In later pregnancy, increased, rather than decreased, levels of ZA2G are observed in pregnancies complicated by preeclampsia^50^. Further, these levels have been positively correlated with blood pressure and renal impairment^50^.

### Comparison with existing prior markers of preeclampsia

Our panel of CMP-associated protein markers achieved a median AUC that is comparable with other suggested biomarkers. Von Daedelzen et al. demonstrated an AUC of 0.88 for a combination of maternal history, symptoms, and clinical lab values^51^. Thangaratinam et al. identified a range of AUC (0.58–0.74) for the prediction of progression to severe preeclampsia based on the patient’s clinical signs and symptoms^52^. The angiogenic markers, sFlt, PlGF, and their ratios, have a range of AUC from 0.52 to 0.74^53,54^. Combining first trimester biomarkers with maternal characteristics and uterine artery Doppler improves prediction with ranges of AUC (0.91–0.96), but requires high levels of technical competence for the performance of Dopplers with adequate reproducibility^55^. Combinations of biomarkers have suggested that an AUC of 0.82 is possible from samples drawn in the first trimester^56^. More recently, using a circulating, small non-coding RNA, a mean AUC of 0.86 has been reported^57^. Even with our limited sample size, our markers are within the range of these other studies. To not over subscribe our data, we intentionally limited our maximum panel size. However, because so many of the individual protein markers reoccurred frequently among the iterated panels, there may be additional information to be extracted and larger panels might improve predictive potential. Future work, with a larger sample size will allow the inclusion of an expanded number of proteins as well as maternal clinical characteristics that will improve the predictive characteristics of these markers. Further, our work suggests the possibility of risk prediction at the end of the first trimester—earlier in gestation than most other biomarker evaluations.

### Clustering of preeclampsia subtypes

In the second portion of this analysis, we present evidence that CMP-associated proteins obtained in the first trimester indicate potential subgroups or subphenotypes among women who go on to develop early preeclampsia < 34 weeks (Table 1). The unsupervised clustering of CMP-associated proteins into two groups demonstrated one group with a more clinically severe presentation associated with higher median 24-h protein loss and higher median maximum systolic and diastolic blood pressures. These more severe preeclampsia-associated characteristics likely prompted the earlier median gestational age at delivery noted in this cluster. Likewise, other metrics of physiologic instability, including an increased likelihood of a creatinine greater than 1 mg/dL, fibrinogen greater than 450 mg/dL, plasma sodium less than 132 Mmol/L, and platelets less than 100,000 per ml, among other physiologic indicators, tended to be associated with this more severe cluster.

### Characteristics of the proteins differentially associated with clusters 1 and 2

Among the proteins and their enriched biological pathways that differed, platelet and coagulation associated pathways were related to Cluster 1 while complement-associated proteins were associated with Cluster 2. Observations regarding CMP platelet degranulation and coagulation pathways associated with PE pathophysiology have been made previously. Multiple reports suggest that CMPs of platelet origin are associated with PE^58,59^. Activated platelets are involved in the remodeling of the spiral arteries and facilitate cytotrophoblastic invasion at the end of the first trimester^60,61^. CMP mediated accumulation of activated platelets in the placental bed has been associated with sterile trophoblastic inflammation^62,63^. Cluster 1, therefore, may reflect preeclampsia pathology associated with aberrant platelet function at the end of the first trimester.

Cluster 2 appears to be associated with pathology linked to complement activity. Complement C2, C3, and C4-A, as well as complement factor H, represent 25% (4/16) of the CMP-associated proteins with higher expression in the second cluster, whereas complement associated-proteins were not present in Cluster 1. Even after controlling for multiple testing, complement C4-A remained significantly associated with Cluster 2. Proteins with functions associated with coagulation (prothrombin, coagulation factor XII, kininogen-1, and heparin cofactor 2) were also associated with this same cluster although only prothrombin remained significant after correction for multiple testing. There is precedent for complement involvement in preeclampsia pathophysiology. Other authors have noted an association between early-onset/severe preeclampsia and complement function^64–66^. Our observations suggest that it may be possible to identify a sub-category of cases of preeclampsia with a dominant complement-associated phenotype at the end of the first trimester. This may offer future potential for screening and therapeutic interventions. There is precedent for such interventions^67^, and several complement inhibitors are currently being evaluated in clinical trials^68^.

The examination of preeclampsia subphenotypes using clustering is a novel and still emerging technique. Leavey et al. utilized placental microarray data to define three sub-etiologies of preeclampsia^4^. They suggest that their clusters include a “maternal” phenotype, represented by term preeclampsia with healthy placentation, a “canonical” phenotype, that exhibits features of severe preeclampsia, and an “immunologic” phenotype suggestive of maternal–fetal incompatibility.

More generally, our analysis suggests that it may be possible to molecularly phenotype cases of preeclampsia early in the disease course. If different underlying pathophysiologic processes lead to the development of different clinical presentations of preeclampsia, then the early identification and specific targeting of these processes may provide a more effective means to prevent (or delay) disease occurrence.

The findings of this investigation must be interpreted within the limitations of its design. As noted above, the sample size limits the number of analytes that can be added into the predictive equations and the number of potential sub-type clusters available to the cluster analysis. The addition of clinical co-factors, such as maternal age and reproductive history, would likely substantially improve the AUC presented. However, such additional information would be at the risk of oversubscribing the data and limiting its ultimate generalizability. Similarly, our analysis of the number of clusters to be examined was, in part, based on sample size. With an increased sample size, more fine grading within cluster types can be explored. Ultimately, both limitations will be addressed with additional analyses of large sample sets. Although we present a sophisticated workflow designed to limit the potential for false discovery, ultimate validation of our findings requires a fully independent validation set. We further acknowledge that we chose to maximize heterogeneity between our cases of early preeclampsia and our term controls. This is colloquially known as a “gap” analysis, and although it is helpful to identify key comparisons, it can also enhance potential findings. True validation of a predictive test will need to include a range of gestational ages among the cases and controls.

This study has many strengths. Characterization of the subjects was performed by a committee of research physicians rather than being abstracted from billing or more generalized data. It is the first to use CMP-associated proteins to stratify the risk of preeclampsia at the end of the first trimester, a comparatively early gestational age. This analysis is also the first to use CMP proteins to attempt “molecular phenotyping” of early preeclampsia and examine substructure within more severe forms of the disease.

## Conclusion

We suggest that CMP-associated proteins, from 10 to 12 weeks of gestation, can be used to stratify the risk of early preeclampsia. Additionally, for those that screen positive, clustering can be used to further characterize the putative subtype of preeclampsia. Eventually, risk stratification and disease phenotype characterizations such as this may be able to optimize prophylactic and therapeutic interventions. As in contemporary cancer therapy, eventually, the treatment chosen would be specified by the patient’s specific molecular signature ascertained in early pregnancy.

## Methods

### Sample collection

We performed a nested case–control study selected from the prospectively collected LIFECODES pregnancy biobank at Brigham and Women’s Hospital, Boston, MA. Patients were approached at their first prenatal visit (median 10.2 weeks gestation). Eligibility criteria included patients who were > 18 years of age, initiated their prenatal care at < 15 weeks of gestation, and planned on delivering at Brigham and Women’s Hospital. After written informed consent was obtained, EDTA plasma samples were obtained, aliquoted, and stored at − 80 degrees centigrade. The biobanking and research protocol were approved by the institutional review board at Brigham and Women’s Hospital. All methods were performed in accordance with relevant guidelines and regulations.

Pregnancy dating was confirmed by ultrasound at ≤ 12 weeks gestation. If consistent with the last menstrual period (LMP) dating, the LMP was used to determine the due date. If not consistent, then the due date was set by the earliest available ultrasound ≤ 12 weeks gestation. Full-term birth was defined as ≥ 37 weeks of gestation. Maternal race was determined by self-identification. The medical record for each subject in the LIFECODES biobank is independently reviewed by two Maternal–Fetal Medicine faculty physicians. Complications and outcomes for each subject are coded using a structured coding tool. The codes from each reviewer are then compared with disagreement in either pregnancy outcome or complication is adjudicated by a review committee. The definition of preeclampsia used by the faculty reviewers is consistent with that of the 2013 Task Force on Hypertension in Pregnancy^69^. In order to maximize clinical relevance and avoid the unintentional picking of “ideal” cases of preeclampsia, the cases were chosen randomly (exclusive of the exclusion criteria noted below) from within our biobank. The cases of preeclampsia presented here represent cases of preeclampsia with severe features^69^. Cases with evidence of hemolysis, and hence suggestive of HELLP syndrome^70,71^, were excluded from this analysis.

We defined exclusion criteria as preexisting medical disorders (preexisting diabetes, current cancer diagnosis, HIV, and hepatitis), and ultrasonically-documented fetal anomalies. The analysis was restricted to singleton gestations. We defined cases as subjects diagnosed with preeclampsia occurring ≤ 35 weeks gestation. Controls were defined as subjects delivering after 37 weeks gestation without evidence of any of the hypertensive diseases of pregnancy (chronic hypertension, gestational hypertension or preeclampsia). Our final sample size consisted of 25 cases that were selected at random from among the 175 subjects with preeclampsia ≤ 35 weeks. Controls were randomly matched 2:1 from among the 2342 subjects meeting the criteria defined above. Cases and controls were matched by maternal age (± 2 years) and gestational age of sampling (± 2 weeks).

### CMP enrichment

We utilized Size Exclusion Chromatography (SEC) for CMP isolation. Our methods have been detailed in our prior publications^26–28^. SEC has been evaluated and reviewed favorably as an efficient means for microparticle isolation^72^. Anonymized EDTA plasma samples identified only by a study number that was agnostic to case or control status were randomly assorted and shipped on dry ice to the David H. Murdock Research Institute (DHMRI, Kannapolis, NC) where CMP-associated protein enrichment was carried out by SEC and isocratically eluted using the NeXosome Elution Reagent. This involved NeXosome Isolation Columns manufactured by AmericanBio, Inc. (Canton, MA). Briefly, these columns were packed by AmericanBio with 2% Sepharose 2B (pore size 60–200 nm) from GE Healthcare Bio-Sciences Corp. (Marlborough, MA) to a total packed volume of 10 mL and delivered to DHMRI under ambient shipping conditions. Once received by DHMRI, the columns were stored at 2–8 °C until use. Prior to using the columns for CMP isolation, they were allowed to equilibrate to room temperature and subsequently washed with NeXosome Elution Regent. EDTA plasma samples were thawed and 1.0 mL of plasma was applied and allowed to incorporate into the NeXosome Isolation Column. The plasma samples were not filtered, diluted, or pretreated prior to application to the columns. Following the incorporation of the sample into the column, the NeXosome Elution Reagent was added and 1.0 mL column fractions were collected. As previously published, the eluted fractions yielded two peaks. The CMPs were captured in the column void volume and resolved from the high abundant soluble protein peak^26^. To minimize the potential for batch effects, the processing of individual samples was performed in random order. Each CMP-containing fraction (0.5 mL aliquots of each fraction) was pooled within each individual sample and a total protein measurement was performed, using the Pierce BCA Protein Assay Kit (ThermoFisher Scientific). An aliquot containing a total protein of 200 µg from each individual CMP isolate pool was then transferred to 2-mL microcentrifuge tubes (VWR, Radnor, PA) and stored at − 80 °C pending completion of all CMP isolate processing. All CMP isolates were then shipped on dry ice to ThermoFisher Scientific’s Biomarker Research Initiatives in Mass Spectrometry (BRIMS) Center (Cambridge, MA) for proteomic analysis.

### Liquid chromatography-mass spectrometry

Study samples were sent on dry ice to the BRIMS institute. Additional aliquots were created and analyzed for system suitability and quality control analysis. These included PRTC standards for system suitability, pooled CMP digests for quality control analysis, and twelve peptide fractions for spectral library creation. Data was acquired via a Vanquish Horizon UHPLC coupled to a Fusion Lumos Orbitrap (ThermoFisher Scientific, Cambridge, MA) mass spectrometer. The spectral library was created on PD 2.1 and searched against Uniprot Human data base^38^. Static cys carboxymethylation, differential deamidation, oxidation, and global proteome profiling were examined. Data were normalized based on TIC.

Briefly, for each sample, a total of 200 ug of CMP protein fraction pool was lyophilized and then dissolved and denatured/reduced with 50 µL 8 M guanidine hydrochloride, 250 mM Tris–HCl, 2.5% n-propanol, 10 mM DTT, pH 8.6 for 1 h at 26ºC. Alkylation of cysteines was performed by adding 4.5 µL of 500 mM sodium iodoacetate and allowing the sample to sit for 2 h. Digestion buffer, 850 µL, 50 mM Tris–HCl 5 mM CaCl_2_, 1% n-propanol, 0.35 mM DTT was added to each tube, and the samples transferred to a 2 mL 96 well ThermoFisher Scientific Polypropylene plate. Pierce TPCK modified trypsin was dissolved in 25 mM acetic acid to a final concentration of 22 mg/mL. To each well was added 250µL of this trypsin solution and the plates were then sealed with a ThermoFisher Scientific Easypeel heat sealing foil. Samples were incubated with shaking at 37 °C for 16 h. Post-digestion, 50 µL of acetic acid was added to each well and the samples lyophilized to dryness for 16 h at 35 mTorr. Each sample was brought up in 450 µL of 2% methanol 1% acetic acid, containing 22.2 fmol/µL of the PRTC peptide mix. An aliquot of each sample 65 µl was transferred to another smaller ThermoFisherScientific plate (AB-1300) and sealed with a ThermoFisher Scientific Easypeel heat sealing foil and refrigerated at 4 °C until use.

### Liquid chromatography

Identical liquid chromatography methods were used for all methods, both library and individual sample quantification runs. Sample plates were loaded onto the UHPLC. The UHPLC system was plumbed with a column compartment divert valve, and a system divert valve. The Vanquish Active solvent preheater at 80 °C is connected to a 2.1 × 50 mm PS-DVB trap column containing 3 µm particles; the column compartment divert valve is then linked to two Acclaim RSLC 120 C18 2.2 µm analytical columns connected in tandem—the compartment housing the analytical columns is held at 60 °C—the analytical columns are, in turn, connected to the system divert valve. Solvent A on the system is Optima LC–MS grade water with 2% methanol and 0.2% formic acid. Solvent B is Optima LC–MS grade water:isopropanol:acetonitrile:formic acid, at a 10:10:80:0.2% ratio. From each well, 45 µL of sample is injected at 1 mL/min, the intra Trap-Analytical column divert column is kept diverted for 1 min to load and desalt the sample, the flow rate is dropped to 250 µL a min and the divert valve switched to the analytical column, and then a gradient of 10–38% B is applied over 50 min, after which the valve is set to divert again, and a 45 µL injection of formic acid is injected while ramping the flow and gradient to 1 mL/min and 100% B. Post-rinsing, the column is equilibrated to A for 6 min at 800 µL/min.

### Library fractionation

Spectral libraries were made by taking 20% of every sample and making a large pool. This pool (2 mg) was injected onto a 4.6 × 250 mm PS-DVB column running a gradient of A 0.2% ammonium hydroxide, 50 mM ammonium formate to B 0.2% ammonium hydroxide. Peptides are fractionated into a 12, 2 mL fractions in a 2.2 mL 96-well plate containing 100µL of 50% acetic acid. Fractions are frozen/lyophilized and dissolved into 250 µL of 2% methanol, 1% acetic acid containing 22.2 fmol/µL of the PRTC peptide mix.

### Mass spectrometry

The Orbitrap was used to generate the peptide spectral libraries and run the individual test samples. Source settings on the system were set to 4.5 kV with a sheath of 25 units, aux of 5 units, and a sweep of 2 units with an ion capillary temperature of 325 and a gas temperature of 275 °C. The source housing drain was removed for this experiment. The Lumos was run in a data-dependent acquisition mode with a “cycle time” limit of 2 s, a full scan mass range of 350–1500 m/z with polysiloxane lock masses being used in the full scan only. For Library acquisition, stepped collision energy ± 5% was used to give better peptide backbone coverage, with a cycle time of 2 s. For the data acquisition on the panel samples, a wide data-dependent acquisition scheme was used. For this setting, a 5-dalton isolation window was used with a 1.2 s cycle time to preserve full scan quantification integrity.

### Database searching and data processing

Raw library files were searched against the IPI human database using SEQUEST with Proteome Discoverer version 2.2. Static carboxymethyl cysteines and dynamic oxidized methionines were used with a parent mass tolerance of 10 ppm and a product ion tolerance of 0.02 Da. Peptides were scored with Percolator and peptides with an FDR of 0.01 or less were used for the libraries.

Signal processing and initial data analysis were carried out using Pinnacle software for multiplexed MRM data analysis (version 1.0.92.0; Optys Tech Corporation). A spectral library was created by importing the Proteome Discoverer MSF files. For global proteome profiling, data were normalized based on total ion chromatograph. Quantified peptides were associated with the protein of origin based on a stratification that included an MSMS dot product score ≥ 0.6. Peptide library retention time tolerance was set to 1 min. The coefficient of variation within each group (cases versus controls) was required to be ≤ 30% and the false discovery rate of the spectral library was ≤ 0.05. Protein concentrations were calculated after normalization to the reference group’s median value. Putative protein targets were identified and ranked by minimizing the coefficient of variation within group and the *p* value associated with the difference between cases and controls. Normalized file areas were then exported for analysis.

### Sample classification

**Figure 5:**
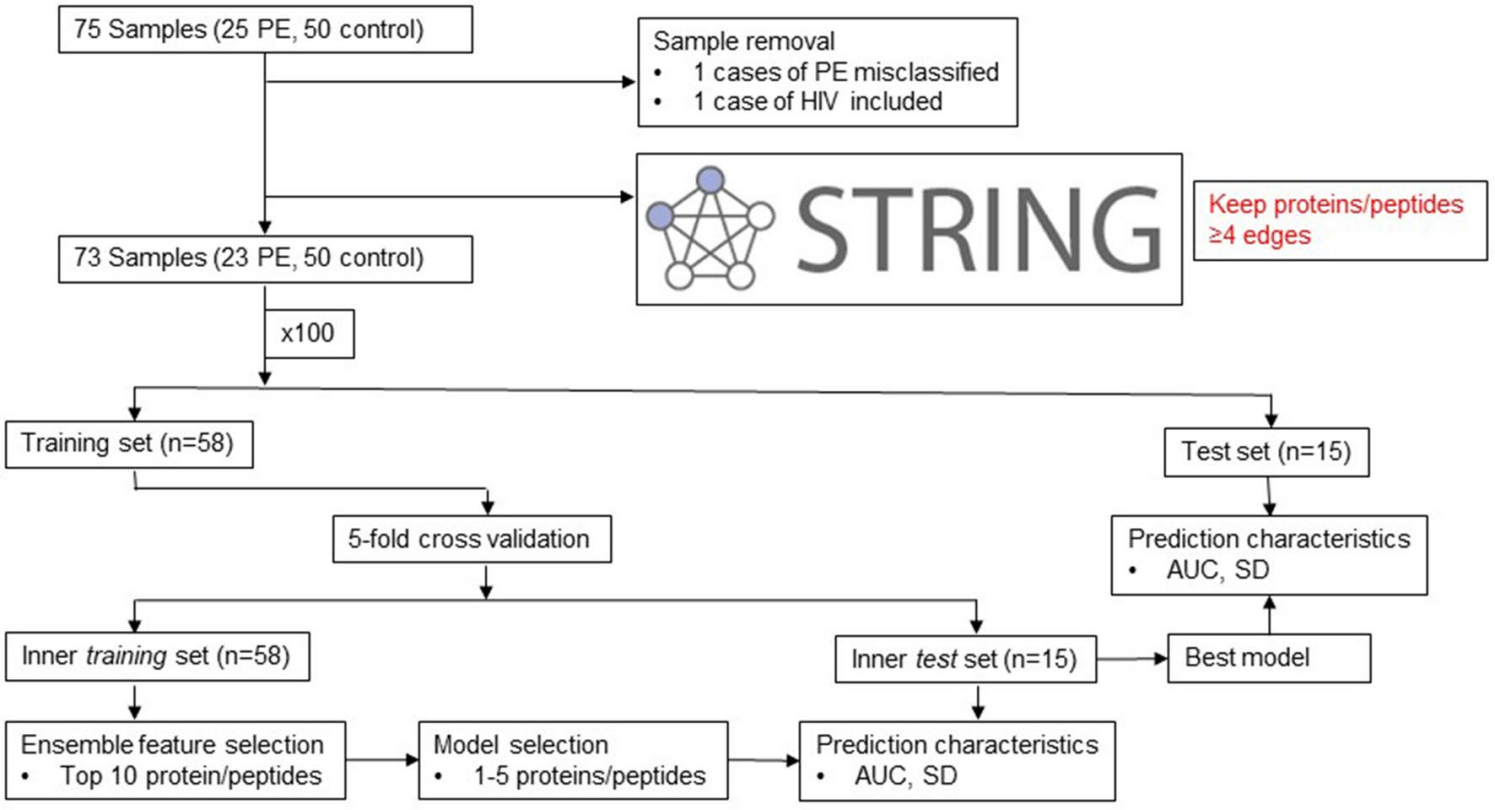
Schematic of workflow for case versus control CMP-associated protein identification.

We initially explored bivariate associations between preeclampsia and individual CMP-associated proteins with significance levels adjusted for multiple testing using the Bonferroni correction. We chose this correction given its conservative tendency^73^. We then sought to establish a panel of CMP-associated proteins that would serve as a classifier for the risk of preeclampsia ≤ 35 weeks. Under the hypothesis that highly correlated analytes are likely to be members of the same biological pathways, we selectively excluded analytes with correlations ≥ 0.7. We adjudicated between two correlated analytes by retaining analytes with a greater number of overall correlations. By retaining the analyte with the greater number of correlations we are assuming that it is a member in a larger number of pathways and hence more functionally important. To further enrich the remaining set of analytes to those with known systematic interactions, we adapted a principle from systems biology that highly interactive elements are more likely to correlate with the state of the overall system and screened the identified proteins for known direct interactions in the STRING database (Fig. 5; www.string-db.org, accessed 5/25/2019)^74,75^. Proteins with four or more edges were retained in the validation procedure. This step aided in the elimination of protein fragments (e.g. hypervariable regions) that represent potential false-positive findings. We termed the data prior to this step the “full” dataset, and that after this step the “restricted” dataset.

To select our putative panel, we chose a cross-validation procedure using logistic regression. As our intention was to create generalizable models and given the limitations of our sample size, the cross-validation procedure was applied over 100 iterations. For each iteration, the sample was randomly divided into a training and validation set (80 vs. 20%). The proteins in the training set were then ranked by their Akaike information criterion (AIC) using an ensemble feature selection procedure that examined the association between each protein and the outcome using a panel of bivariate tests^76^. The top 10 individual proteins in each iteration were selected. The training set was subjected to fivefold cross-validation. Accordingly, the training set was divided into 5 subsets of equal size. The elements of each subset were unique, and a sample could belong to only one subset per iteration. Four of the subsets were then combined and used to train a logistic regression on the fifth set. This was repeated five times such that each set functioned in the test role only once.

The *glmulti* package was used to evaluate all sets of predictors and rank each model by AIC^77^. To avoid overfitting, given the limited sample size, the model was restricted to no more than 4 predictors. The model with the greatest area under the curve (AUC) and the lowest standard deviation of the AUC was then tested against the set-aside, external validation set. The AUC and standard deviation of the AUC of this external validation set was then recorded for the 100 iterations. The models were then ranked according to these parameters. The ranked models and the frequency with which individual proteins occurred in the overall models were recorded.

To confirm the utility of the workflow described above, we randomly permuted the case versus control labels of the samples. The workflow was re-run as described above. Predictive statistics for the observed versus permuted data were then graphically compared (Fig. 5). The logic of this permutation was adapted from that of Yoffe et al.^57^.

### Preeclampsia sub-classification

To explore potential subphenotypes within preeclampsia, we performed an unsupervised clustering analysis only among the cases from the restricted dataset. We used the Average Silhouette method^78,79^ to determine the appropriate number of clusters within the preeclampsia cases. K-means clustering was then used to partition the cases into the appropriate number of clusters^80^. Both the Average Silhouette method and K-means were run using Euclidean space.

All statistical analyses were performed in the R computing environment (version 3.2.5; https://www.r-project.org). To obtain biological insight into the functional role of the identified proteins, we used g:Profiler package in R for the pathway enrichment analysis, tissue enrichment and identification of the associated Gene Ontology (GO) terms^37^. Functional information on individual proteins was obtained from the UniProt database^38^.

## Supplementary information

Supplementary Tables.
