# Supplementary material for: Late first trimester circulating microparticle proteins predict the risk of preeclampsia < 35 weeks and suggest phenotypic differences among affected cases
Source: Sci Rep. 2020 Oct 21;10:17353. doi: 10.1038/s41598-020-74078-w (PMC7578826; doi:10.1038/s41598-020-74078-w)
Supplement: Supplementary file 1 — Supplementary Tables. [file 41598_2020_74078_MOESM1_ESM.docx]

**Late First Trimester Circulating Microparticle Proteins Predict the Risk of Preeclampsia <35 Weeks and Suggest Phenotypic Differences Among Affected Cases**

Thomas F. McElrath, MD, PhD(a)^*^, David E. Cantonwine, PhD, MPH(a), Kathryn J. Gray, MD, PhD(a), Hooman Mirzakhani, MD, PhD, MMSc(b), Robert C. Doss, PhD(c), Najmuddin Khaja, PhD(c), Malik Khalid(c), Gail Page(c), Brian Brohman(c), Zhen Zhang, PhD(d), David Sarracino, PhD(f) Kevin P. Rosenblatt, MD, PhD(c,e)

(a) Division of Maternal-Fetal Medicine, Department of Obstetrics and Gynecology, Brigham and Women’s Hospital, Boston, MA

(b) Department of Medicine, Channing Division of Network Medicine, Brigham and Women's Hospital, Boston, MA

(c) NX Prenatal Inc. Louisville, KY

(d) Center for Biomarker Discovery and Translation, Department of Pathology, Johns Hopkins University School of Medicine, Baltimore, MD

(e) Division of Oncology, Department of Internal Medicine, University of Texas Health Science Center at Houston, McGovern Medical School, Houston, TX

(f) Thermo Fisher Scientific, Cambridge, Massachusetts

**Supplementary Information:**

**Table S1:** Proteins Identified Post Signal Processing

| **Name** | **Full Name** |
| --- | --- |
| A0A024R8D8_HUMAN | Progestagen-associated endometrial protein (Placental protein 14, pregnancy-associated endometrial alpha-2-globulin, alpha uterine protein) |
| E9PQG4_HUMAN | Myomegalin |
| PAEP_HUMAN | sp\|P09466\|PAEP_HUMAN Glycodelin OS=Homo sapiens GN=PAEP PE=1 SV=2 |
| C6K6H8_HUMAN | MHC class I antigen |
| A2NWW3_HUMAN | VH-3 family (VH26)D/J protein (Fragment) |
| I6Y0B1_HUMAN | MHC class I antigen (Fragment) |
| JPH1_HUMAN | Junctophilin-1 |
| A2MG_HUMAN | Alpha-2-macroglobulin |
| A5YM46_HUMAN | ERN2 protein |
| B2RB52_HUMAN | cDNA, FLJ95314, highly similar to Homo sapiens transducin (beta)-like 2 (TBL2), transcript variant 1 |
| B4DG07_HUMAN | cDNA FLJ58159, highly similar to RAB6-interacting protein 2 |
| B2R950_HUMAN | cDNA, FLJ94213, highly similar to Homo sapiens pregnancy-zone protein (PZP), mRNA |
| PAPP1_HUMAN | Pappalysin-1 |
| KAD2_HUMAN | Adenylate kinase 2, mitochondrial |
| Q4KMY3_HUMAN | C10orf28 protein |
| PRG4_HUMAN | Proteoglycan 4 |
| PEDF_HUMAN | Pigment epithelium-derived factor |
| KAIN_HUMAN | Kallistatin OS=Homo sapiens |
| CO5_HUMAN | Complement C5 |
| B2RCQ9_HUMAN | cDNA, FLJ96225, highly similar to Homo sapiens heat shock 70kDa protein 1-like (HSPA1L) |
| F5H7M0_HUMAN | SKI/DACH domain-containing protein 1 OS=Homo sapiens |
| LAC7_HUMAN | Ig lambda-7 chain C region OS=Homo sapiens |
| F13A_HUMAN | Coagulation factor XIII A chain |
| LV106_HUMAN | Ig lambda chain V-I region WAH |
| Q5NV91_HUMAN | V2-19 protein (Fragment) |
| TPC11_HUMAN | Trafficking protein particle complex subunit 11 |
| B3KR56_HUMAN | cDNA FLJ33721 fis, clone BRAWH2016792, highly similar to Angiomotin-like protein 2 |
| A2N0U6_HUMAN | VH6DJ protein |
| Q9UL75_HUMAN | Myosin-reactive immunoglobulin heavy chain variable region (Fragment) |
| GP1BA_HUMAN | Platelet glycoprotein Ib alpha chain |
| Q5NV73_HUMAN | V2-13 protein (Fragment) |
| PRG2_HUMAN | Bone marrow proteoglycan |
| CFAI_HUMAN | Complement factor I |
| Q6P5S3_HUMAN | Uncharacterized protein |
| A0A5E4_HUMAN | Uncharacterized protein |
| A2NUT2_HUMAN | Lambda-chain (AA -20 to 215) |
| Q6GMV8_HUMAN | Uncharacterized protein |
| Q7Z2U7_HUMAN | Uncharacterized protein |
| Q8NEJ1_HUMAN | Uncharacterized protein |
| S6BGD6_HUMAN | IgG L chain |
| B2R6L0_HUMAN | Tubulin beta chain |
| HV304_HUMAN | Ig heavy chain V-III region TIL |
| PLEC_HUMAN | Plectin |
| Q5NV88_HUMAN | V1-22 protein (Fragment) |
| HV301_HUMAN | Ig heavy chain V-III region TRO |
| APOC2_HUMAN | Apolipoprotein C-II |
| Q9UL88_HUMAN | Myosin-reactive immunoglobulin heavy chain variable region (Fragment) |
| ZA2G_HUMAN | Zinc-alpha-2-glycoprotein |
| A0A075B6K3_HUMAN | Protein IGLV2-11 (Fragment) |
| FCN2_HUMAN | Ficolin-2 |
| Q5NV74_HUMAN | V2-14 protein (Fragment) |
| A0A075B6R2_HUMAN | Protein IGHV4-4 (Fragment) |
| LCAT_HUMAN | Phosphatidylcholine-sterol acyltransferase |
| Q5T985_HUMAN | Inter-alpha-trypsin inhibitor heavy chain H2 |
| APOC4_HUMAN | Apolipoprotein C-IV |
| AMBP_HUMAN | Protein AMBP |
| V9HWB4_HUMAN | Epididymis secretory sperm binding protein Li 89n |
| IGHG4_HUMAN | Ig gamma-4 chain C region |
| O95973_HUMAN | VH4 heavy chain variable region (Fragment) |
| Q6MZU6_HUMAN | Putative uncharacterized protein DKFZp686C15213 |
| Q6N093_HUMAN | Putative uncharacterized protein DKFZp686I04196 (Fragment) |
| A0A075B6R9_HUMAN | Protein IGKV2D-24 (Fragment) |
| Q9Y509_HUMAN | VH3 protein (Fragment) |
| E9KL26_HUMAN | Epididymis tissue protein Li 173 |
| F2VNE4_HUMAN | MHC class I antigen (Fragment) |
| H0Y447_HUMAN | Cytidine and dCMP deaminase domain-containing protein 1 (Fragment) |
| LV103_HUMAN | Ig lambda chain V-I region NEW |
| K7ESF8_HUMAN | Zinc finger protein 235 |
| TM163_HUMAN | Transmembrane protein 163 |
| HEP2_HUMAN | Heparin cofactor 2 |
| J7RT51_HUMAN | MHC class I antigen (Fragment) |
| A1L179_HUMAN | KIF5C protein (Fragment) |
| Q5XTR9_HUMAN | Hemoglobin delta-beta fusion protein (Fragment) |
| TETN_HUMAN | Tetranectin |
| SLK_HUMAN | STE20-like serine/threonine-protein kinase |
| C1RL_HUMAN | Complement C1r subcomponent-like protein |
| D2KZ31_HUMAN | MHC class I antigen (Fragment) |
| APOC3_HUMAN | Apolipoprotein C-III |
| CFAH_HUMAN | Complement factor H |
| CO4A_HUMAN | Complement C4-A |
| Q8NF17_HUMAN | FLJ00385 protein (Fragment) |
| KV302_HUMAN | Ig kappa chain V-III region SIE |
| KV305_HUMAN | Ig kappa chain V-III region WOL |
| A1AG2_HUMAN | Alpha-1-acid glycoprotein 2 |
| A2AP_HUMAN | Alpha-2-antiplasmin |
| A6NIW5_HUMAN | Peroxiredoxin 2, isoform CRA_a |
| KV309_HUMAN | Ig kappa chain V-III region VG (Fragment) |
| TSP4_HUMAN | Thrombospondin-4 |
| HV103_HUMAN | Ig heavy chain V-I region V35 |
| KV304_HUMAN | Ig kappa chain V-III region Ti |
| KV307_HUMAN | Ig kappa chain V-III region GOL |
| A8K008_HUMAN | cDNA FLJ78387 |
| Q6MZQ6_HUMAN | Putative uncharacterized protein DKFZp686G11190 |
| Q6MZV7_HUMAN | Putative uncharacterized protein DKFZp686C11235 |
| Q6N089_HUMAN | Putative uncharacterized protein DKFZp686P15220 |
| Q6N094_HUMAN | Putative uncharacterized protein DKFZp686O01196 |
| Q6N095_HUMAN | Putative uncharacterized protein DKFZp686K03196 |
| Q6N096_HUMAN | Putative uncharacterized protein DKFZp686I15196 |
| Q7Z351_HUMAN | Putative uncharacterized protein DKFZp686N02209 |
| S6B291_HUMAN | IgG H chain |
| V9HW68_HUMAN | Epididymis luminal protein 214 |
| APOE_HUMAN | Apolipoprotein E |
| CO2_HUMAN | Complement C2 |
| CAPS1_HUMAN | Calcium-dependent secretion activator 1 |
| Q5NV90_HUMAN | V2-17 protein (Fragment) |
| HEMO_HUMAN | Hemopexin |
| H0YDW7_HUMAN | CD44 antigen (Fragment) |
| HV107_HUMAN | Ig heavy chain V-I region Mot |
| B2R5S1_HUMAN | cDNA, FLJ92595, highly similar to Homo sapiens angiotensinogen (serine (or cysteine) proteinaseinhibitor, clade A (alpha-1 antiproteinase, antitrypsin), member 8)(AGT), mRNA |
| Q59EP2_HUMAN | Angiotensinogen variant (Fragment) |
| VTNC_HUMAN | Vitronectin |
| A1AT_HUMAN | Alpha-1-antitrypsin |
| IGHG2_HUMAN | Ig gamma-2 chain C region |
| HV303_HUMAN | Ig heavy chain V-III region VH26 |
| IGHD_HUMAN | Ig delta chain C region |
| A2NWW1_HUMAN | VH-3 family (VH26)D/J protein (Fragment) |
| A2NWW5_HUMAN | VH-3 family (VH26.)D/J protein (Fragment) |
| A2NWX0_HUMAN | VH-3 family (VH26)D/J protein (Fragment) |
| Q9UL93_HUMAN | Myosin-reactive immunoglobulin heavy chain variable region (Fragment) |
| A2JA14_HUMAN | Anti-mucin1 heavy chain variable region (Fragment) |
| Q0ZCI9_HUMAN | Immunglobulin heavy chain variable region (Fragment) |
| APOH_HUMAN | Beta-2-glycoprotein 1 |
| TRIPB_HUMAN | Thyroid receptor-interacting protein 11 |
| Q8WYY6_HUMAN | Putative uncharacterized protein |
| Q96QL5_HUMAN | PSG4 protein |
| IGJ_HUMAN | Immunoglobulin J chain |
| HV309_HUMAN | Ig heavy chain V-III region NIE |
| A2RU29_HUMAN | MORC3 protein |
| GP1BB_HUMAN | Platelet glycoprotein Ib beta chain |
| KV122_HUMAN | Ig kappa chain V-I region BAN |
| SAA4_HUMAN | Serum amyloid A-4 protein |
| A0A075B6K1_HUMAN | Protein IGLV2-14 (Fragment) |
| FA12_HUMAN | Coagulation factor XII |
| H0YL22_HUMAN | Complex I intermediate-associated protein 30, mitochondrial |
| Q584P1_HUMAN | Putative uncharacterized protein PCYOX1 (Fragment) |
| KNG1_HUMAN | Kininogen-1 |
| F5H1U9_HUMAN | Multiple PDZ domain protein |
| CD5L_HUMAN | CD5 antigen-like |
| VWA3A_HUMAN | von Willebrand factor A domain-containing protein 3A |
| E7EVP7_HUMAN | Inositol 1,4,5-trisphosphate receptor type 1 |
| CRP_HUMAN | C-reactive protein |
| TBB4A_HUMAN | Tubulin beta-4A chain |
| TBB4B_HUMAN | Tubulin beta-4B chain |
| APOL1_HUMAN | Apolipoprotein L1 |
| A5PL32_HUMAN | APOL1 protein (Fragment) |
| A0A075B7E8_HUMAN | Protein IGHV3OR16-13 (Fragment) |
| HV302_HUMAN | Ig heavy chain V-III region WEA |
| HV306_HUMAN | Ig heavy chain V-III region BUT |
| K1C9_HUMAN | Keratin, type I cytoskeletal 9 |
| A2J1M5_HUMAN | Rheumatoid factor RF-IP4 (Fragment) |
| A2NWX4_HUMAN | VH-7 family (N54P3)D/J protein (Fragment) |
| A0A075B6I1_HUMAN | Protein IGLV4-60 (Fragment) |
| J3KN23_HUMAN | Protein lin-7 homolog A |
| Q15403_HUMAN | Pregnancy-specific protein (Fragment) |
| PIGR_HUMAN | Polymeric immunoglobulin receptor |
| TRFE_HUMAN | Serotransferrin |
| G8JLD3_HUMAN | ELKS/Rab6-interacting/CAST family member 1 |
| G3XAC3_HUMAN | Breast cancer 1, early onset, isoform CRA_l |
| HPT_HUMAN | Haptoglobin |
| HPTR_HUMAN | Haptoglobin-related protein |
| MTEF3_HUMAN | Transcription termination factor 3, mitochondrial |
| LUM_HUMAN | Lumican |
| KV105_HUMAN | Ig kappa chain V-I region DEE |
| CO3_HUMAN | Complement C3 |
| KV121_HUMAN | Ig kappa chain V-I region Ni |
| KV303_HUMAN | Ig kappa chain V-III region NG9 (Fragment) |
| A2MYD6_HUMAN | V1-2 protein (Fragment) |
| LV211_HUMAN | Ig lambda chain V-II region NIG-84 |
| Q65ZC9_HUMAN | Single-chain Fv (Fragment) |
| Q9BR60_HUMAN | OS9 protein (Fragment) |
| A0A075B6H9_HUMAN | Protein IGLV4-69 (Fragment) |
| THRB_HUMAN | sp\|P00734\|THRB_HUMAN Prothrombin OS=Homo sapiens GN=F2 PE=1 SV=2 |
| K2C1_HUMAN | Keratin, type II cytoskeletal 1 |
| GP112_HUMAN | Probable G-protein coupled receptor 112 |
| C1R_HUMAN | Complement C1r subcomponent |
| ZN251_HUMAN | Zinc finger protein 251 |
| H0Y713_HUMAN | Peroxisome proliferator-activated receptor gamma coactivator 1-beta (Fragment) |
| APOD_HUMAN | Apolipoprotein D |
| J3KNQ2_HUMAN | Fibronectin type III domain-containing protein 1 (Fragment) |
| PLMN_HUMAN | Plasminogen |
| Q9UL72_HUMAN | Myosin-reactive immunoglobulin heavy chain variable region (Fragment) |
| A0A024R1N1_HUMAN | Myosin, heavy polypeptide 9, non-muscle, isoform CRA_a |
| A0A075B7B8_HUMAN | Protein IGHV3OR16-12 (Fragment) |
| E1Y7G1_HUMAN | MHC class I antigen (Fragment) |
| FCGBP_HUMAN | IgGFc-binding protein |
| C5MK54_HUMAN | MHC class I antigen |
| SYCY1_HUMAN | Syncytin-1 |
| DYH3_HUMAN | Dynein heavy chain 3, axonemal |
| ANT3_HUMAN | Antithrombin-III |
| PGRP2_HUMAN | N-acetylmuramoyl-L-alanine amidase |
| ALBU_HUMAN | Serum albumin |
| K22E_HUMAN | Keratin, type II cytoskeletal 2 epidermal |
| Q9UL86_HUMAN | Myosin-reactive immunoglobulin kappa chain variable region (Fragment) |
| HRG_HUMAN | Histidine-rich glycoprotein |
| B2R8I2_HUMAN | cDNA, FLJ93914, highly similar to Homo sapiens histidine-rich glycoprotein (HRG), mRNA |
| TSP1_HUMAN | Thrombospondin-1 |
| A2MYD5_HUMAN | V2-6 protein (Fragment) |
| B4E1Z4_HUMAN | Uncharacterized protein |
| MBL2_HUMAN | Mannose-binding protein C |
| F6IQR0_HUMAN | MHC class I antigen (Fragment) |
| PLF4_HUMAN | Platelet factor 4 |
| AACT_HUMAN | Alpha-1-antichymotrypsin |
| B2R5G8_HUMAN | Serum amyloid A protein |
| CO9_HUMAN | Complement component C9 |
| B4DK28_HUMAN | cDNA FLJ56000, highly similar to Carboxypeptidase-like protein X2 |
| KV113_HUMAN | Ig kappa chain V-I region Lay OS=Homo sapiens PE=1 SV=1 |
| KV306_HUMAN | Ig kappa chain V-III region POM |
| KV308_HUMAN | Ig kappa chain V-III region CLL |
| ECM1_HUMAN | Extracellular matrix protein 1 |
| TIKI1_HUMAN | Metalloprotease TIKI1 |
| SEPP1_HUMAN | Selenoprotein P |
| PHLD_HUMAN | Phosphatidylinositol-glycan-specific phospholipase D |
| TBB5_HUMAN | Tubulin beta chain |
| IGHM_HUMAN | Ig mu chain C region |
| MUCB_HUMAN | Ig mu heavy chain disease protein |
| C1K0X8_HUMAN | MHC class I antigen |
| C5IWX6_HUMAN | MHC class I antigen |
| F6IQK2_HUMAN | MHC class I antigen (Fragment) |
| F6IQT3_HUMAN | MHC class I antigen (Fragment) |
| Q8MHM5_HUMAN | MHC class I antigen (Fragment) |
| S5DN70_HUMAN | MHC class I antigen (Fragment) |
| Q4G1H0_HUMAN | Abnormal spindle-like microcephaly associated splice variant 2 |
| ANGT_HUMAN | Angiotensinogen |
| C1QC_HUMAN | Complement C1q subcomponent subunit C |
| Q15402_HUMAN | Pregnancy-specific protein (Fragment) |
| MMRN1_HUMAN | Multimerin-1 |

**Table S2.** Functional and placental enrichment of proteins/genes distinguishing cases of preeclampsia from controls.

| Enrichment | Enriched Term | GO-Term ID | Number of gene/proteins in Term ID | Number of genes/proteins | Number of Intersection | Corrected  P-value | Intersections |
| --- | --- | --- | --- | --- | --- | --- | --- |
| **Biological Process** | | | | | | | |
| GO:BP | negative regulation of blood coagulation | GO:0030195 | 52 | 17 | 4 | 0.000236024 | GP1BA,VTN,APOH,THBS1 |
| GO:BP | negative regulation of hemostasis | GO:1900047 | 53 | 17 | 4 | 0.000255143 | GP1BA,VTN,APOH,THBS1 |
| GO:BP | negative regulation of coagulation | GO:0050819 | 58 | 17 | 4 | 0.000368599 | GP1BA,VTN,APOH,THBS1 |
| GO:BP | negative regulation of wound healing | GO:0061045 | 78 | 17 | 4 | 0.001224915 | GP1BA,VTN,APOH,THBS1 |
| GO:BP | regulation of blood coagulation | GO:0030193 | 80 | 17 | 4 | 0.001356571 | GP1BA,VTN,APOH,THBS1 |
| GO:BP | regulation of hemostasis | GO:1900046 | 81 | 17 | 4 | 0.001426212 | GP1BA,VTN,APOH,THBS1 |
| GO:BP | regulation of coagulation | GO:0050818 | 87 | 17 | 4 | 0.001901456 | GP1BA,VTN,APOH,THBS1 |
| GO:BP | negative regulation of response to external stimulus | GO:0032102 | 417 | 17 | 6 | 0.002471203 | GP1BA,VTN,APOH,C5,THBS1,APOD |
| GO:BP | negative regulation of response to wounding | GO:1903035 | 93 | 17 | 4 | 0.002485467 | GP1BA,VTN,APOH,THBS1 |
| GO:BP | fibrinolysis | GO:0042730 | 27 | 17 | 3 | 0.003306406 | GP1BA,APOH,THBS1 |
| GO:BP | negative regulation of endopeptidase activity | GO:0010951 | 253 | 17 | 5 | 0.004702427 | VTN,C5,SERPIND1,SERPINA3,THBS1 |
| GO:BP | negative regulation of peptidase activity | GO:0010466 | 264 | 17 | 5 | 0.005791244 | VTN,C5,SERPIND1,SERPINA3,THBS1 |
| GO:BP | smooth muscle cell-matrix adhesion | GO:0061302 | 5 | 17 | 2 | 0.013660809 | VTN,APOD |
| GO:BP | wound healing/restoration of injured tissue | GO:0042060 | 584 | 17 | 6 | 0.017224167 | GP1BA,VTN,APOH,SERPIND1,THBS1,APOD |
| GO:BP | regulation of wound healing | GO:0061041 | 156 | 17 | 4 | 0.01947961 | GP1BA,VTN,APOH,THBS1 |
| GO:BP | blood coagulation | GO:0007596 | 340 | 17 | 5 | 0.01982643 | GP1BA,VTN,APOH,SERPIND1,THBS1 |
| GO:BP | hemostasis | GO:0007599 | 345 | 17 | 5 | 0.021276776 | GP1BA,VTN,APOH,SERPIND1,THBS1 |
| GO:BP | coagulation | GO:0050817 | 348 | 17 | 5 | 0.022186058 | GP1BA,VTN,APOH,SERPIND1,THBS1 |
| GO:BP | negative regulation of proteolysis | GO:0045861 | 363 | 17 | 5 | 0.027199229 | VTN,C5,SERPIND1,SERPINA3,THBS1 |
| GO:BP | zymogen activation | GO:0031638 | 55 | 17 | 3 | 0.02917265 | C1RL,APOH,THBS1 |
| GO:BP | complement activation | GO:0006956 | 178 | 17 | 4 | 0.032755465 | VTN,C1RL,C5,MBL2 |
| GO:BP | lipid transport | GO:0006869 | 390 | 17 | 5 | 0.03841954 | APOC2,APOH,THBS1,APOD,LCAT |
| GO:BP | regulation of response to wounding | GO:1903034 | 189 | 17 | 4 | 0.041450234 | GP1BA,VTN,APOH,THBS1 |
| GO:BP | response to wounding | GO:0009611 | 702 | 17 | 6 | 0.048968104 | GP1BA,VTN,APOH,SERPIND1,THBS1,APOD |
| **Molecular Function** | | | | | | | |
| GO:MF | lipoprotein lipase activator activity | GO:0060230 | 4 | 17 | 2 | 0.001305802 | APOC2,APOH |
| GO:MF | heparin binding | GO:0008201 | 171 | 17 | 4 | 0.004351715 | VTN,APOH,SERPIND1,THBS1 |
| GO:MF | glycosaminoglycan binding | GO:0005539 | 237 | 17 | 4 | 0.015611267 | VTN,APOH,SERPIND1,THBS1 |
| GO:MF | sulfur compound binding | GO:1901681 | 253 | 17 | 4 | 0.020119598 | VTN,APOH,SERPIND1,THBS1 |
| GO:MF | lipase activator activity | GO:0060229 | 15 | 17 | 2 | 0.022713244 | APOC2,APOH |
| **Cellular Component** | | | | | | | |
| GO:CC | extracellular matrix | GO:0031012 | 534 | 17 | 7 | 3.57073E-05 | GP1BA,VTN,AZGP1,APOH,MBL2,SERPINA3,THBS1 |
| GO:CC | extracellular exosome | GO:0070062 | 2144 | 17 | 11 | 4.31184E-05 | GP1BA,VTN,C1RL,AZGP1,APOH,C5,SERPIND1,SERPINA3,THBS1,APOD,LCAT |
| GO:CC | extracellular vesicle | GO:1903561 | 2167 | 17 | 11 | 4.81412E-05 | GP1BA,VTN,C1RL,AZGP1,APOH,C5,SERPIND1,SERPINA3,THBS1,APOD,LCAT |
| GO:CC | extracellular organelle | GO:0043230 | 2172 | 17 | 11 | 4.93001E-05 | GP1BA,VTN,C1RL,AZGP1,APOH,C5,SERPIND1,SERPINA3,THBS1,APOD,LCAT |
| GO:CC | extracellular space | GO:0005615 | 3543 | 17 | 13 | 6.63237E-05 | GP1BA,VTN,C1RL,AZGP1,APOC2,APOH,C5,SERPIND1,MBL2,SERPINA3,THBS1,APOD,LCAT |
| GO:CC | collagen-containing extracellular matrix | GO:0062023 | 407 | 17 | 6 | 0.000164913 | VTN,AZGP1,APOH,MBL2,SERPINA3,THBS1 |
| GO:CC | vesicle | GO:0031982 | 3914 | 17 | 13 | 0.000221874 | GP1BA,VTN,C1RL,AZGP1,APOC2,APOH,C5,SERPIND1,SERPINA3,THBS1,CADPS,APOD,LCAT |
| GO:CC | high-density lipoprotein particle | GO:0034364 | 27 | 17 | 3 | 0.000293764 | APOC2,APOH,LCAT |
| GO:CC | lipoprotein particle | GO:1990777 | 37 | 17 | 3 | 0.000776021 | APOC2,APOH,LCAT |
| GO:CC | plasma lipoprotein particle | GO:0034358 | 37 | 17 | 3 | 0.000776021 | APOC2,APOH,LCAT |
| GO:CC | protein-lipid complex | GO:0032994 | 39 | 17 | 3 | 0.000911732 | APOC2,APOH,LCAT |
| GO:CC | extracellular region | GO:0005576 | 4548 | 17 | 13 | 0.001338873 | GP1BA,VTN,C1RL,AZGP1,APOC2,APOH,C5,SERPIND1,MBL2,SERPINA3,THBS1,APOD,LCAT |
| GO:CC | chylomicron | GO:0042627 | 14 | 17 | 2 | 0.011568273 | APOC2,APOH |
| GO:CC | triglyceride-rich plasma lipoprotein particle | GO:0034385 | 21 | 17 | 2 | 0.026597096 | APOC2,APOH |
| GO:CC | very-low-density lipoprotein particle | GO:0034361 | 21 | 17 | 2 | 0.026597096 | APOC2,APOH |
| **Human Protein Atlas** | | | | | | | |
| HPA | placenta; decidual cells | HPA:035010_10 | 1166 | 4 | 4 | 0.02683443 | VTN,C1RL,SERPIND1,GP1BA |
| HPA | cervix, uterine; squamous epithelial cells | HPA:009020_10 | 1355 | 6 | 5 | 0.04031129 | GP1BA,VTN,C1RL,APOC2,SERPIND1 |
| HPA | testis; cells in seminiferous ducts | HPA:053020_10 | 1392 | 6 | 5 | 0.045962943 | GP1BA,VTN,C1RL,APOC2,SERPIND1 |
| HPA | cervix, uterine; glandular cells | HPA:009010_10 | 1402 | 6 | 5 | 0.047592607 | GP1BA,VTN,C1RL,APOC2,SERPIND1 |
| HPA | soft tissue 1; fibroblasts | HPA:047030_10 | 1405 | 6 | 5 | 0.048090273 | GP1BA,VTN,C1RL,APOC2,SERPIND1 |
| HPA | lymph node; germinal center cells | HPA:028010_10 | 1409 | 6 | 5 | 0.048760191 | GP1BA,VTN,C1RL,APOC2,SERPIND1 |
| HPA | nasopharynx; respiratory epithelial cells | HPA:029010_10 | 1410 | 6 | 5 | 0.048928813 | GP1BA,VTN,C1RL,APOC2,SERPIND1 |
| HPA | appendix; lymphoid tissue | HPA:002020_10 | 1415 | 6 | 5 | 0.049778823 | GP1BA,VTN,C1RL,APOC2,SERPIND1 |

**Table S3.** Functional and placental enrichment of proteins/genes distinguishing preeclampsia cases (Cluster 1).

| Enrichment | Enriched Term | GO-Term ID | Number of gene/proteins in Term ID | Number of genes/proteins in cluster 1 | Number of Intersection | Corrected  P-value | Intersections |
| --- | --- | --- | --- | --- | --- | --- | --- |
| **Biological Processes** | | | | | | | |
| GO:BP | platelet degranulation | GO:0002576 | 126 | 8 | 3 | 0.0228668 | ECM1,A2M,VWF |
| GO:BP | blood coagulation, intrinsic pathway | GO:0007597 | 18 | 8 | 2 | 0.032689 | A2M,VWF |
| Molecular Function |  |  |  |  |  |  |  |
| GO:MF | protease binding | GO:0002020 | 131 | 8 | 3 | 0.00306256 | ECM1,A2M,VWF |
| GO:MF | extracellular matrix structural constituent | GO:0005201 | 165 | 8 | 3 | 0.00610551 | ECM1,PRG2,VWF |
| GO:MF | immunoglobulin binding | GO:0019865 | 23 | 8 | 2 | 0.00652396 | JCHAIN,VWF |
| GO:MF | signaling receptor binding | GO:0005102 | 1625 | 8 | 5 | 0.03904176 | JCHAIN,ECM1,A2M,IGLL1,VWF |
| **Cellular Component** | | | | | | | |
| GO:CC | collagen-containing extracellular matrix | GO:0062023 | 405 | 8 | 4 | 0.00161759 | ECM1,PRG2,A2M,VWF |
| GO:CC | extracellular matrix | GO:0031012 | 530 | 8 | 4 | 0.00465902 | ECM1,PRG2,A2M,VWF |
| GO:CC | secretory granule | GO:0030141 | 835 | 8 | 4 | 0.02733119 | ECM1,PRG2,A2M,VWF |
| GO:CC | secretory granule lumen | GO:0034774 | 319 | 8 | 3 | 0.02973638 | ECM1,A2M,VWF |
| GO:CC | cytoplasmic vesicle lumen | GO:0060205 | 323 | 8 | 3 | 0.03084789 | ECM1,A2M,VWF |
| GO:CC | vesicle lumen | GO:0031983 | 325 | 8 | 3 | 0.03141365 | ECM1,A2M,VWF |
| HPA | Not enriched | - | - | - | - | - | - |

**Table S4** Functional and placental enrichment of proteins/genes distinguishing preeclampsia cases (Cluster 2).

| Enrichment | Enriched Term | GO-Term ID | Number of gene/proteins in Term ID | Number of genes/proteins in cluster 2 | Number of Intersection | Corrected  P-value | Intersections |
| --- | --- | --- | --- | --- | --- | --- | --- |
| **Biological Processes** | | | | | | | |
| GO:BP | regulation of humoral immune response | GO:0002920 | 129 | 22 | 10 | 2.65E-13 | VTN,C4A,F2,C3,C2,CFH,HPX,CD5L,CLU,C5 |
| GO:BP | regulation of complement activation | GO:0030449 | 110 | 22 | 9 | 6.90E-12 | VTN,C4A,F2,C3,C2,CFH,CD5L,CLU,C5 |
| GO:BP | complement activation | GO:0006956 | 178 | 22 | 10 | 7.10E-12 | VTN,C4A,F2,C3,C2,CFH,CRP,CD5L,CLU,C5 |
| GO:BP | humoral immune response | GO:0006959 | 362 | 22 | 11 | 1.92E-10 | VTN,C4A,F2,C3,C2,CFH,CRP,HPX,CD5L,CLU,C5 |
| GO:BP | regulation of proteolysis | GO:0030162 | 737 | 22 | 12 | 1.61E-08 | VTN,SERPINF1,C4A,F2,C3,AGT,GPLD1,F12,KNG1,SERPIND1,CLU,C5 |
| GO:BP | regulation of immune effector process | GO:0002697 | 448 | 22 | 10 | 7.16E-08 | VTN,C4A,F2,C3,C2,CFH,HPX,CD5L,CLU,C5 |
| GO:BP | positive regulation of immune response | GO:0050778 | 894 | 22 | 12 | 1.52E-07 | VTN,C4A,F2,C3,C2,GPLD1,CFH,CRP,HPX,CD5L,CLU,C5 |
| GO:BP | proteolysis | GO:0006508 | 1807 | 22 | 15 | 1.57E-07 | VTN,SERPINF1,C4A,F2,ENSG00000244255,C3,AGT,C2,GPLD1,F12,KNG1,SERPIND1, CD5L,CLU,C5 |
| GO:BP | positive regulation of immune system process | GO:0002684 | 1189 | 22 | 13 | 2.18E-07 | VTN,C4A,F2,C3,C2,GPLD1,CFH,CRP,HPX,CD5L,THBS4,CLU,C5 |
| GO:BP | negative regulation of proteolysis | GO:0045861 | 361 | 22 | 9 | 3.27E-07 | VTN,SERPINF1,C4A,F2,C3,AGT,KNG1,SERPIND1,C5 |
| GO:BP | activation of immune response | GO:0002253 | 724 | 22 | 11 | 3.44E-07 | VTN,C4A,F2,C3,C2,GPLD1,CFH,CRP,CD5L,CLU,C5 |
| GO:BP | negative regulation of endopeptidase activity | GO:0010951 | 252 | 22 | 8 | 6.15E-07 | VTN,SERPINF1,C4A,C3,AGT,KNG1,SERPIND1,C5 |
| GO:BP | inflammatory response | GO:0006954 | 765 | 22 | 11 | 6.17E-07 | SERPINF1,C4A,F2,C3,AGT,F12,CRP,KNG1,CD5L,CLU,C5 |
| GO:BP | response to external stimulus | GO:0009605 | 2887 | 22 | 17 | 6.37E-07 | VTN,SERPINF1,C4A,F2,C3,AGT,C2,F12,CFH,CRP,KNG1,SERPIND1,HPX,THBS4,CLU,C5,ALB |
| GO:BP | humoral immune response mediated by circulating immunoglobulin | GO:0002455 | 155 | 22 | 7 | 8.02E-07 | C4A,C3,C2,CRP,HPX,CLU,C5 |
| GO:BP | negative regulation of peptidase activity | GO:0010466 | 264 | 22 | 8 | 8.89E-07 | VTN,SERPINF1,C4A,C3,AGT,KNG1,SERPIND1,C5 |
| GO:BP | defense response | GO:0006952 | 1760 | 22 | 14 | 1.88349E-06 | SERPINF1,C4A,F2,C3,AGT,C2,F12,CFH,CRP,KNG1,HPX,CD5L,CLU,C5 |
| GO:BP | regulation of immune response | GO:0050776 | 1113 | 22 | 12 | 1.90004E-06 | VTN,C4A,F2,C3,C2,GPLD1,CFH,CRP,HPX,CD5L,CLU,C5 |
| GO:BP | negative regulation of hydrolase activity | GO:0051346 | 465 | 22 | 9 | 3.04164E-06 | VTN,SERPINF1,C4A,C3,AGT,KNG1,SERPIND1,C5,APOC3 |
| GO:BP | immunoglobulin mediated immune response | GO:0016064 | 222 | 22 | 7 | 9.83966E-06 | C4A,C3,C2,CRP,HPX,CLU,C5 |
| GO:BP | B cell mediated immunity | GO:0019724 | 225 | 22 | 7 | 1.07989E-05 | C4A,C3,C2,CRP,HPX,CLU,C5 |
| GO:BP | response to stress | GO:0006950 | 4085 | 22 | 18 | 1.28852E-05 | VTN,SERPINF1,C4A,F2,C3,AGT,C2,F12,CFH,CRP,KNG1,SERPIND1,HPX,CD5L,THBS4,CLU,C5,ALB |
| GO:BP | regulation of immune system process | GO:0002682 | 1671 | 22 | 13 | 1.45219E-05 | VTN,C4A,F2,C3,C2,GPLD1,CFH,CRP,HPX,CD5L,THBS4,CLU,C5 |
| GO:BP | positive regulation of response to stimulus | GO:0048584 | 2514 | 22 | 15 | 1.69198E-05 | VTN,C4A,F2,C3,AGT,C2,GPLD1,F12,CFH,CRP,HPX,CD5L,THBS4,CLU,C5 |
| GO:BP | complement activation, classical pathway | GO:0006958 | 142 | 22 | 6 | 2.47244E-05 | C4A,C3,C2,CRP,CLU,C5 |
| GO:BP | regulation of endopeptidase activity | GO:0052548 | 429 | 22 | 8 | 4.00899E-05 | VTN,SERPINF1,C4A,C3,AGT,KNG1,SERPIND1,C5 |
| GO:BP | regulation of response to stimulus | GO:0048583 | 4370 | 22 | 18 | 4.02328E-05 | VTN,SERPINF1,C4A,F2,C3,AGT,C2,GPLD1,F12,CFH,CRP,KNG1,HPX,CD5L,THBS4,CLU,C5,APOC3 |
| GO:BP | regulation of peptidase activity | GO:0052547 | 456 | 22 | 8 | 6.4337E-05 | VTN,SERPINF1,C4A,C3,AGT,KNG1,SERPIND1,C5 |
| GO:BP | immune effector process | GO:0002252 | 1273 | 22 | 11 | 0.000127312 | VTN,C4A,F2,C3,C2,CFH,CRP,HPX,CD5L,CLU,C5 |
| GO:BP | positive regulation of apoptotic cell clearance | GO:2000427 | 8 | 22 | 3 | 0.000146255 | C4A,C3,C2 |
| GO:BP | positive regulation of transport | GO:0051050 | 998 | 22 | 10 | 0.000160963 | VTN,C4A,F2,C3,AGT,C2,GPLD1,CADPS,CLU,C5 |
| GO:BP | lymphocyte mediated immunity | GO:0002449 | 354 | 22 | 7 | 0.000242357 | C4A,C3,C2,CRP,HPX,CLU,C5 |
| GO:BP | adaptive immune response based on somatic recombination of immune receptors built from immunoglobulin superfamily domains | GO:0002460 | 358 | 22 | 7 | 0.000261583 | C4A,C3,C2,CRP,HPX,CLU,C5 |
| GO:BP | vesicle-mediated transport | GO:0016192 | 2146 | 22 | 13 | 0.000296687 | VTN,C4A,F2,C3,C2,CADPS,CRP,KNG1,HPX,CD5L,CLU,APOC3,ALB |
| GO:BP | complement-dependent cytotoxicity | GO:0097278 | 10 | 22 | 3 | 0.000312903 | C3,CFH,CD5L |
| GO:BP | negative regulation of catalytic activity | GO:0043086 | 824 | 22 | 9 | 0.000425532 | VTN,SERPINF1,C4A,C3,AGT,KNG1,SERPIND1,C5,APOC3 |
| GO:BP | regulation of apoptotic cell clearance | GO:2000425 | 11 | 22 | 3 | 0.000429899 | C4A,C3,C2 |
| GO:BP | regulation of response to external stimulus | GO:0032101 | 1122 | 22 | 10 | 0.000481854 | VTN,SERPINF1,F2,C3,AGT,F12,KNG1,HPX,THBS4,C5 |
| GO:BP | regulation of cellular protein metabolic process | GO:0032268 | 2698 | 22 | 14 | 0.000487681 | VTN,SERPINF1,C4A,F2,C3,AGT,GPLD1,F12,KNG1,SERPIND1,HPX,THBS4,CLU,C5 |
| GO:BP | protein metabolic process | GO:0019538 | 5905 | 22 | 19 | 0.000589444 | VTN,SERPINF1,C4A,F2,ENSG00000244255,C3,AGT,C2,GPLD1,F12,KNG1,SERPIND1,HPX,CD5L,THBS4,CLU,C5,APOC3,ALB |
| GO:BP | immune response | GO:0006955 | 2286 | 22 | 13 | 0.000628183 | VTN,C4A,F2,C3,C2,GPLD1,F12,CFH,CRP,HPX,CD5L,CLU,C5 |
| GO:BP | negative regulation of blood coagulation | GO:0030195 | 52 | 22 | 4 | 0.00072713 | VTN,F2,F12,KNG1 |
| GO:BP | negative regulation of hemostasis | GO:1900047 | 53 | 22 | 4 | 0.000785852 | VTN,F2,F12,KNG1 |
| GO:BP | negative regulation of protein metabolic process | GO:0051248 | 1186 | 22 | 10 | 0.000806929 | VTN,SERPINF1,C4A,F2,C3,AGT,KNG1,SERPIND1,CLU,C5 |
| GO:BP | regulation of transport | GO:0051049 | 1915 | 22 | 12 | 0.000837816 | VTN,C4A,F2,C3,AGT,C2,GPLD1,CADPS,CRP,CLU,C5,APOC3 |
| GO:BP | defense response to other organism | GO:0098542 | 1196 | 22 | 10 | 0.000872228 | C4A,F2,C3,C2,F12,CFH,CRP,HPX,CLU,C5 |
| GO:BP | complement activation, alternative pathway | GO:0006957 | 14 | 22 | 3 | 0.000946112 | C3,CFH,C5 |
| GO:BP | regulation of protein metabolic process | GO:0051246 | 2870 | 22 | 14 | 0.001067648 | VTN,SERPINF1,C4A,F2,C3,AGT,GPLD1,F12,KNG1,SERPIND1,HPX,THBS4,CLU,C5 |
| GO:BP | negative regulation of coagulation | GO:0050819 | 58 | 22 | 4 | 0.00113402 | VTN,F2,F12,KNG1 |
| GO:BP | regulation of localization | GO:0032879 | 2893 | 22 | 14 | 0.001180814 | VTN,SERPINF1,C4A,F2,C3,AGT,C2,GPLD1,CADPS,CRP,THBS4,CLU,C5,APOC3 |
| GO:BP | regulation of hydrolase activity | GO:0051336 | 1289 | 22 | 10 | 0.001741196 | VTN,SERPINF1,C4A,C3,AGT,GPLD1,KNG1,SERPIND1,C5,APOC3 |
| GO:BP | blood coagulation, intrinsic pathway | GO:0007597 | 18 | 22 | 3 | 0.002114188 | F2,F12,KNG1 |
| GO:BP | innate immune response | GO:0045087 | 1000 | 22 | 9 | 0.002171373 | C4A,C3,C2,F12,CFH,CRP,HPX,CLU,C5 |
| GO:BP | receptor-mediated endocytosis | GO:0006898 | 319 | 22 | 6 | 0.002941258 | VTN,C3,HPX,CLU,APOC3,ALB |
| GO:BP | negative regulation of wound healing | GO:0061045 | 76 | 22 | 4 | 0.003379715 | VTN,F2,F12,KNG1 |
| GO:BP | regulation of blood coagulation | GO:0030193 | 79 | 22 | 4 | 0.003948424 | VTN,F2,F12,KNG1 |
| GO:BP | regulation of hemostasis | GO:1900046 | 80 | 22 | 4 | 0.004152878 | VTN,F2,F12,KNG1 |
| GO:BP | immune system process | GO:0002376 | 3214 | 22 | 14 | 0.004409647 | VTN,C4A,F2,C3,C2,GPLD1,F12,CFH,CRP,HPX,CD5L,THBS4,CLU,C5 |
| GO:BP | regulation of vesicle-mediated transport | GO:0060627 | 558 | 22 | 7 | 0.005141192 | VTN,C4A,C3,C2,CADPS,CLU,APOC3 |
| GO:BP | negative regulation of cellular protein metabolic process | GO:0032269 | 1118 | 22 | 9 | 0.005484428 | VTN,SERPINF1,C4A,F2,C3,AGT,KNG1,SERPIND1,C5 |
| GO:BP | import into cell | GO:0098657 | 819 | 22 | 8 | 0.00552072 | VTN,C3,AGT,HPX,CD5L,CLU,APOC3,ALB |
| GO:BP | regulation of coagulation | GO:0050818 | 86 | 22 | 4 | 0.005549011 | VTN,F2,F12,KNG1 |
| GO:BP | post-translational protein modification | GO:0043687 | 363 | 22 | 6 | 0.006206914 | C4A,C3,GPLD1,KNG1,SERPIND1,ALB |
| GO:BP | negative regulation of response to wounding | GO:1903035 | 90 | 22 | 4 | 0.006655356 | VTN,F2,F12,KNG1 |
| GO:BP | positive regulation of phosphate metabolic process | GO:0045937 | 1147 | 22 | 9 | 0.006774734 | VTN,F2,C3,AGT,GPLD1,HPX,THBS4,CLU,C5 |
| GO:BP | positive regulation of phosphorus metabolic process | GO:0010562 | 1147 | 22 | 9 | 0.006774734 | VTN,F2,C3,AGT,GPLD1,HPX,THBS4,CLU,C5 |
| GO:BP | regulation of catalytic activity | GO:0050790 | 2338 | 22 | 12 | 0.007244659 | VTN,SERPINF1,C4A,F2,C3,AGT,GPLD1,KNG1,SERPIND1,CLU,C5,APOC3 |
| GO:BP | blood coagulation, fibrin clot formation | GO:0072378 | 27 | 22 | 3 | 0.007524147 | F2,F12,KNG1 |
| GO:BP | protein activation cascade | GO:0072376 | 27 | 22 | 3 | 0.007524147 | F2,F12,KNG1 |
| GO:BP | organonitrogen compound metabolic process | GO:1901564 | 6854 | 22 | 19 | 0.007991397 | VTN,SERPINF1,C4A,F2,ENSG00000244255,C3,AGT,C2,GPLD1,F12,KNG1,SERPIND1,HPX,CD5L,THBS4,CLU,C5,APOC3,ALB |
| GO:BP | plasma lipoprotein particle remodeling | GO:0034369 | 28 | 22 | 3 | 0.008420316 | AGT,APOC3,ALB |
| GO:BP | protein-lipid complex remodeling | GO:0034368 | 28 | 22 | 3 | 0.008420316 | AGT,APOC3,ALB |
| GO:BP | negative regulation of molecular function | GO:0044092 | 1178 | 22 | 9 | 0.008437016 | VTN,SERPINF1,C4A,C3,AGT,KNG1,SERPIND1,C5,APOC3 |
| GO:BP | response to other organism | GO:0051707 | 1536 | 22 | 10 | 0.008601367 | C4A,F2,C3,C2,F12,CFH,CRP,HPX,CLU,C5 |
| GO:BP | response to external biotic stimulus | GO:0043207 | 1538 | 22 | 10 | 0.008702904 | C4A,F2,C3,C2,F12,CFH,CRP,HPX,CLU,C5 |
| GO:BP | leukocyte mediated immunity | GO:0002443 | 874 | 22 | 8 | 0.008943235 | C4A,F2,C3,C2,CRP,HPX,CLU,C5 |
| GO:BP | positive regulation of reactive oxygen species metabolic process | GO:2000379 | 97 | 22 | 4 | 0.008973752 | F2,AGT,CRP,CLU |
| GO:BP | protein-containing complex remodeling | GO:0034367 | 29 | 22 | 3 | 0.009384392 | AGT,APOC3,ALB |
| GO:BP | response to biotic stimulus | GO:0009607 | 1561 | 22 | 10 | 0.009948238 | C4A,F2,C3,C2,F12,CFH,CRP,HPX,CLU,C5 |
| GO:BP | regulation of plasma lipoprotein particle levels | GO:0097006 | 105 | 22 | 4 | 0.012300739 | AGT,GPLD1,APOC3,ALB |
| GO:BP | negative regulation of response to external stimulus | GO:0032102 | 410 | 22 | 6 | 0.012483164 | VTN,SERPINF1,F2,F12,KNG1,C5 |
| GO:BP | regulation of biological quality | GO:0065008 | 4103 | 22 | 15 | 0.013212489 | VTN,SERPINF1,F2,C3,AGT,GPLD1,F12,CADPS,CRP,KNG1,SERPIND1,HPX,CLU,APOC3,ALB |
| GO:BP | regulation of receptor-mediated endocytosis | GO:0048259 | 107 | 22 | 4 | 0.0132581 | VTN,C3,CLU,APOC3 |
| GO:BP | positive regulation of cellular protein metabolic process | GO:0032270 | 1625 | 22 | 10 | 0.014269906 | VTN,F2,C3,AGT,GPLD1,F12,HPX,THBS4,CLU,C5 |
| GO:BP | acute inflammatory response | GO:0002526 | 110 | 22 | 4 | 0.014795936 | F2,C3,F12,CRP |
| GO:BP | adaptive immune response | GO:0002250 | 658 | 22 | 7 | 0.015205867 | C4A,C3,C2,CRP,HPX,CLU,C5 |
| GO:BP | positive regulation of biological process | GO:0048518 | 6290 | 22 | 18 | 0.016221862 | VTN,SERPINF1,C4A,F2,C3,AGT,C2,GPLD1,F12,CFH,CADPS,CRP,KNG1,HPX,CD5L,THBS4,CLU,C5 |
| GO:BP | endocytosis | GO:0006897 | 669 | 22 | 7 | 0.016944865 | VTN,C3,HPX,CD5L,CLU,APOC3,ALB |
| GO:BP | negative regulation of triglyceride catabolic process | GO:0010897 | 5 | 22 | 2 | 0.023343737 | GPLD1,APOC3 |
| GO:BP | positive regulation of protein metabolic process | GO:0051247 | 1730 | 22 | 10 | 0.024939894 | VTN,F2,C3,AGT,GPLD1,F12,HPX,THBS4,CLU,C5 |
| GO:BP | cytolysis | GO:0019835 | 40 | 22 | 3 | 0.025152376 | F2,GPLD1,C5 |
| GO:BP | regulation of triglyceride metabolic process | GO:0090207 | 41 | 22 | 3 | 0.027116425 | C3,GPLD1,APOC3 |
| GO:BP | negative regulation of cellular metabolic process | GO:0031324 | 2660 | 22 | 12 | 0.028292355 | VTN,SERPINF1,C4A,F2,C3,AGT,GPLD1,KNG1,SERPIND1,CLU,C5,APOC3 |
| GO:BP | positive regulation of protein phosphorylation | GO:0001934 | 1025 | 22 | 8 | 0.028817856 | VTN,F2,C3,AGT,HPX,THBS4,CLU,C5 |
| GO:BP | plasma lipoprotein particle organization | GO:0071827 | 45 | 22 | 3 | 0.035980754 | AGT,APOC3,ALB |
| GO:BP | positive regulation of phosphorylation | GO:0042327 | 1077 | 22 | 8 | 0.041277482 | VTN,F2,C3,AGT,HPX,THBS4,CLU,C5 |
| GO:BP | regulation of multicellular organismal process | GO:0051239 | 3300 | 22 | 13 | 0.043254982 | VTN,SERPINF1,F2,C3,AGT,GPLD1,F12,CRP,KNG1,THBS4,CLU,C5,APOC3 |
| GO:BP | protein-lipid complex subunit organization | GO:0071825 | 49 | 22 | 3 | 0.046567666 | AGT,APOC3,ALB |
| GO:BP | apoptotic cell clearance | GO:0043277 | 49 | 22 | 3 | 0.046567666 | C4A,C3,C2 |
| GO:BP | regulation of high-density lipoprotein particle clearance | GO:0010982 | 7 | 22 | 2 | 0.048948632 | GPLD1,APOC3 |
| **Molecular Function** | | | | | | | |
| GO:MF | endopeptidase inhibitor activity | GO:0004866 | 177 | 22 | 7 | 3.51E-07 | SERPINF1,C4A,C3,AGT,KNG1,SERPIND1,C5 |
| GO:MF | endopeptidase regulator activity | GO:0061135 | 184 | 22 | 7 | 4.61E-07 | SERPINF1,C4A,C3,AGT,KNG1,SERPIND1,C5 |
| GO:MF | peptidase inhibitor activity | GO:0030414 | 184 | 22 | 7 | 4.61E-07 | SERPINF1,C4A,C3,AGT,KNG1,SERPIND1,C5 |
| GO:MF | peptidase regulator activity | GO:0061134 | 221 | 22 | 7 | 1.6484E-06 | SERPINF1,C4A,C3,AGT,KNG1,SERPIND1,C5 |
| GO:MF | enzyme inhibitor activity | GO:0004857 | 380 | 22 | 8 | 2.6624E-06 | SERPINF1,C4A,C3,AGT,KNG1,SERPIND1,C5,APOC3 |
| GO:MF | heparin binding | GO:0008201 | 170 | 22 | 6 | 1.2705E-05 | VTN,F2,CFH,KNG1,SERPIND1,THBS4 |
| GO:MF | glycosaminoglycan binding | GO:0005539 | 236 | 22 | 6 | 8.8674E-05 | VTN,F2,CFH,KNG1,SERPIND1,THBS4 |
| GO:MF | sulfur compound binding | GO:1901681 | 250 | 22 | 6 | 0.00012442 | VTN,F2,CFH,KNG1,SERPIND1,THBS4 |
| GO:MF | serine-type endopeptidase activity | GO:0004252 | 165 | 22 | 5 | 0.00042238 | F2,ENSG00000244255,C2,F12,CD5L |
| GO:MF | enzyme regulator activity | GO:0030234 | 1037 | 22 | 9 | 0.00049786 | SERPINF1,C4A,F2,C3,AGT,KNG1,SERPIND1,C5,APOC3 |
| GO:MF | serine-type peptidase activity | GO:0008236 | 186 | 22 | 5 | 0.00076156 | F2,ENSG00000244255,C2,F12,CD5L |
| GO:MF | molecular function regulator | GO:0098772 | 1827 | 22 | 11 | 0.00081108 | SERPINF1,C4A,F2,C3,AGT,GPLD1,KNG1,SERPIND1,THBS4,C5,APOC3 |
| GO:MF | hydrolase activity, acting on acid phosphorus-nitrogen bonds | GO:0016825 | 191 | 22 | 5 | 0.00086739 | F2,ENSG00000244255,C2,F12,CD5L |
| GO:MF | serine hydrolase activity | GO:0017171 | 191 | 22 | 5 | 0.00086739 | F2,ENSG00000244255,C2,F12,CD5L |
| GO:MF | lipoprotein particle receptor binding | GO:0070325 | 26 | 22 | 3 | 0.00122107 | CRP,CLU,APOC3 |
| GO:MF | signaling receptor binding | GO:0005102 | 1625 | 22 | 10 | 0.00239525 | VTN,F2,C3,AGT,CRP,KNG1,THBS4,CLU,C5,APOC3 |
| GO:MF | complement component C1q binding | GO:0001849 | 8 | 22 | 2 | 0.01206215 | C4A,CRP |
| GO:MF | opsonin binding | GO:0001846 | 15 | 22 | 2 | 0.0450004 | C4A,CRP |
| GO:MF | endopeptidase activity | GO:0004175 | 439 | 22 | 5 | 0.04713748 | F2,ENSG00000244255,C2,F12,CD5L |
| **Cellular Component** | | | | | | | |
| GO:CC | blood microparticle | GO:0072562 | 141 | 22 | 11 | 3.72E-16 | VTN,C4A,F2,C3,AGT,CFH,KNG1,HPX,CD5L,CLU,ALB |
| GO:CC | extracellular exosome | GO:0070062 | 2143 | 22 | 18 | 9.04E-12 | VTN,SERPINF1,C4A,F2,C3,AGT,C2,GPLD1,F12,CFH,KNG1,SERPIND1,HPX,THBS4,CLU,C5,APOC3,ALB |
| GO:CC | extracellular vesicle | GO:1903561 | 2166 | 22 | 18 | 1.09E-11 | VTN,SERPINF1,C4A,F2,C3,AGT,C2,GPLD1,F12,CFH,KNG1,SERPIND1,HPX,THBS4,CLU,C5,APOC3,ALB |
| GO:CC | extracellular organelle | GO:0043230 | 2171 | 22 | 18 | 1.14E-11 | VTN,SERPINF1,C4A,F2,C3,AGT,C2,GPLD1,F12,CFH,KNG1,SERPIND1,HPX,THBS4,CLU,C5,APOC3,ALB |
| GO:CC | extracellular space | GO:0005615 | 3541 | 22 | 20 | 9.21E-11 | VTN,SERPINF1,C4A,F2,C3,AGT,C2,GPLD1,F12,CFH,CRP,KNG1,SERPIND1,HPX,CD5L,THBS4,CLU,C5,APOC3,ALB |
| GO:CC | extracellular region part | GO:0044421 | 3751 | 22 | 20 | 2.85E-10 | VTN,SERPINF1,C4A,F2,C3,AGT,C2,GPLD1,F12,CFH,CRP,KNG1,SERPIND1,HPX,CD5L,THBS4,CLU,C5,APOC3,ALB |
| GO:CC | extracellular region | GO:0005576 | 4536 | 22 | 21 | 3.44E-10 | VTN,SERPINF1,C4A,F2,C3,AGT,C2,GPLD1,F12,CFH,CRP,KNG1,SERPIND1,HPX,CD5L,THBS4,CLU,VWA3A,C5,APOC3,ALB |
| GO:CC | extracellular matrix | GO:0031012 | 530 | 22 | 11 | 8.56E-10 | VTN,SERPINF1,F2,AGT,GPLD1,F12,KNG1,HPX,THBS4,CLU,APOC3 |
| GO:CC | collagen-containing extracellular matrix | GO:0062023 | 405 | 22 | 10 | 1.98E-09 | VTN,SERPINF1,F2,AGT,F12,KNG1,HPX,THBS4,CLU,APOC3 |
| GO:CC | vesicle | GO:0031982 | 3891 | 22 | 19 | 1.52E-08 | VTN,SERPINF1,C4A,F2,C3,AGT,C2,GPLD1,F12,CFH,CADPS,KNG1,SERPIND1,HPX,THBS4,CLU,C5,APOC3,ALB |
| GO:CC | endoplasmic reticulum lumen | GO:0005788 | 306 | 22 | 7 | 7.8571E-06 | C4A,F2,C3,KNG1,SERPIND1,CLU,ALB |
| GO:CC | endoplasmic reticulum | GO:0005783 | 1925 | 22 | 10 | 0.00502707 | VTN,C4A,F2,C3,F12,KNG1,SERPIND1,THBS4,CLU,ALB |
| GO:CC | spherical high-density lipoprotein particle | GO:0034366 | 9 | 22 | 2 | 0.00951405 | CLU,APOC3 |
| GO:CC | platelet alpha granule lumen | GO:0031093 | 66 | 22 | 3 | 0.01230132 | KNG1,CLU,ALB |
| GO:CC | platelet alpha granule | GO:0031091 | 90 | 22 | 3 | 0.03101328 | KNG1,CLU,ALB |
| **Human Placenta Atlas** | | | | | | | |
| HPA | placenta; trophoblastic cells | HPA:035020_10 | 1473 | 13 | 8 | 0.04996393 | VTN,AGT,C2,CFH,CRP,SERPIND1,HPX,ALB |
| HPA | cervix, uterine; glandular cells | HPA:009010_10 | 1402 | 13 | 8 | 0.03492448 | VTN,AGT,C2,CFH,CRP,SERPIND1,HPX,ALB |

**Table S5.** Population Characteristics of Preeclampsia Cluster 1 and Cluster 2

| **Characteristic** | **Cluster 1**  **(N=7)** | **Cluster 2**  **(N=16)** | **p-value^a^** |
| --- | --- | --- | --- |
|  | Median (IQR) or N (%) | Median (IQR) or N (%) |  |
| Maternal age | 28.6 (25-36.6) | 32.9 (29.4-36.7) | 0.59 |
| Maternal race |  |  | 0.39 |
| Black | 1 (14.3%) | 5 (31.3%) |  |
| Hispanic | 0 (0.0%) | 2 (12.5%) |  |
| White, non-Hispanic | 5 (71.4%) | 10 (62.5%) |  |
| Nullipara | 1 (14.3%) | 4 (25.0%) | 0.57 |
| Smoking | 1 (14.3%) | 3 (19.0%) | 0.79 |
| Married | 6 (85.7%) | 10 (62.5%) | 0.27 |
|  |  |  |  |
| Chronic hypertension | 1 (14.3%) | 2 (12.5%) | 0.82 |
| Pre pregnancy BMI | 28.3 (24.7-30.8) | 23.6 (20.5-28.7) | 0.23 |
| PE in prior pregnancy | 2 (28.6%) | 4 (25.0%) | 0.71 |
| Prior spontaneous PTB | 4 (25.0%) | 8 (22.2%) | 0.89 |
|  |  |  |  |
| Gestational age at: |  |  |  |
| sampling | 11.2 (8.5-13.7) | 11.4 (9.8-13.5) | 0.92 |
| delivery | 35.3 (33.8-35.4) | 34.1 (29.9-35.6) | 0.14 |
|  |  |  |  |
| Birth metrics (in g): |  |  |  |
| Birth weight | 2339 (1956-2800) | 2168 (1181-2675) | 0.46 |
| Z-score | -0.41 (-1.3-0.5) | -0.20 (-0.9-0.6) | 0.53 |
|  |  |  |  |
| Infant gender: |  |  |  |
| female | 4 (57.1%) | 11 (68.8%) | 0.59 |
| male | 3 (42.1%) | 5 (31.3%) |  |
| ^a^P-values calculated with Wilcoxon Rank Sum test, ANOVA, Chi Square test, or Fisher Exact test where appropriate | | | |
